# Targeted inhibition of PTPN22 is a novel approach to alleviate osteogenic responses in aortic valve interstitial cells and aortic valve lesions in mice

**DOI:** 10.1186/s12916-023-02888-6

**Published:** 2023-07-13

**Authors:** Shunyi Li, Zichao Luo, Shuwen Su, Liming Wen, Gaopeng Xian, Jing Zhao, Xingbo Xu, Dingli Xu, Qingchun Zeng

**Affiliations:** 1 State Key Laboratory of Organ Failure Research, Department of Cardiology, Nanfang Hospital, Southern Medical University, Guangzhou, 510515 China; 2 Guangdong Provincial Key Laboratory of Cardiac Function and Microcirculation, Southern Medical University, Guangzhou, 510515 China; 3 State Key Laboratory of Quality Research in Chinese Medicine, Institute of Chinese Medical Sciences, University of Macau, Macau, China; 4 Department of Cardiology and Pneumology, University Medical Center of Göttingen, Georg-August-University, Göttingen, Germany

**Keywords:** Calcific aortic valve disease, PTPN22, Natural product, Osteogenic responses, Mitochondrial stress

## Abstract

**Background:**

Calcific aortic valve disease (CAVD) is the most prevalent valvular disease and has high morbidity and mortality. CAVD is characterized by complex pathophysiological processes, including inflammation-induced osteoblastic differentiation in aortic valve interstitial cells (AVICs). Novel anti-CAVD agents are urgently needed. Protein tyrosine phosphatase nonreceptor type 22 (PTPN22), an intracellular nonreceptor-like protein tyrosine phosphatase, is involved in several chronic inflammatory diseases, including rheumatoid arthritis and diabetes. However, it is unclear whether PTPN22 is involved in the pathogenesis of CAVD.

**Methods:**

We obtained the aortic valve tissue from human and cultured AVICs from aortic valve. We established CAVD mice model by wire injury. Transcriptome sequencing, western bolt, qPCR, and immunofluorescence were performed to elucidate the molecular mechanisms.

**Results:**

Here, we determined that PTPN22 expression was upregulated in calcific aortic valve tissue, AVICs treated with osteogenic medium, and a mouse model of CAVD. In vitro, overexpression of PTPN22 induced osteogenic responses, whereas siRNA-mediated PTPN22 knockdown abolished osteogenic responses and mitochondrial stress in the presence of osteogenic medium. In vivo, PTPN22 ablation ameliorated aortic valve lesions in a wire injury-induced CAVD mouse model, validating the pathogenic role of PTPN22 in CAVD. Additionally, we discovered a novel compound, 13-hydroxypiericidin A 10-O-α-D-glucose (1 → 6)-β-D-glucoside (S18), in a marine-derived Streptomyces strain that bound to PTPN22 with high affinity and acted as a novel inhibitor. Incubation with S18 suppressed osteogenic responses and mitochondrial stress in human AVICs induced by osteogenic medium. In mice with aortic valve injury, S18 administration markedly alleviated aortic valve lesions.

**Conclusion:**

PTPN22 plays an essential role in the progression of CAVD, and inhibition of PTPN22 with S18 is a novel option for the further development of potent anti-CAVD drugs.

**Graphical Abstract:**

Therapeutic inhibition of PTPN22 retards aortic valve calcification through modulating mitochondrial dysfunction in AVICs.

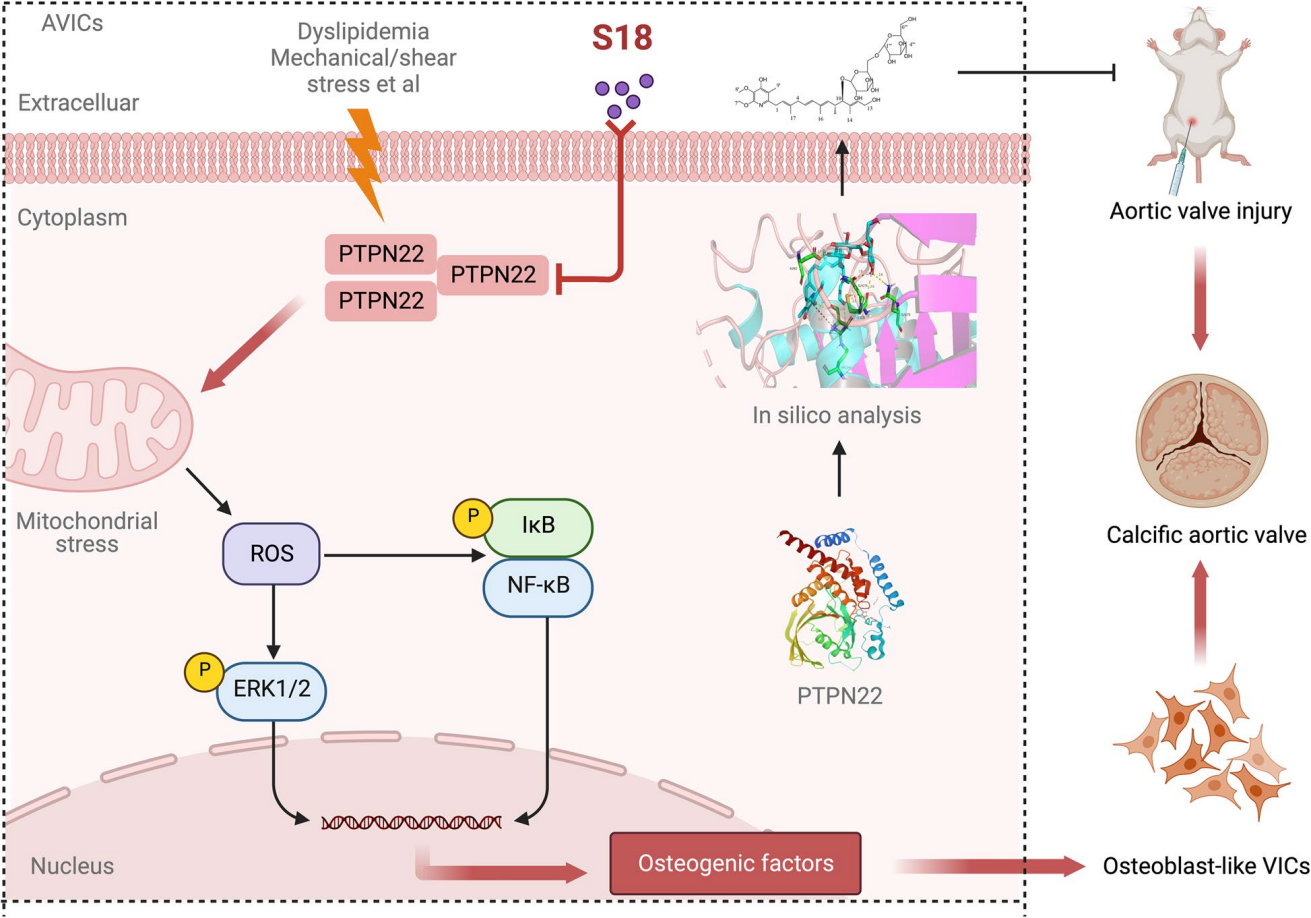

**Supplementary Information:**

The online version contains supplementary material available at 10.1186/s12916-023-02888-6.

## Background

Calcific aortic valve disease (CAVD) is the most prevalent valvular disease in individuals over 65 years of age. Progressive aortic valve calcification will develop into aortic stenosis, and 82.7% of deaths in individuals with aortic valve disease in developed countries are attributed to aortic stenosis [1–3]. To date, there is no pharmacological therapy for retarding CAVD progression, and valve replacement remains the sole treatment for CAVD patients in the advanced stage [4]. Discovering potential targets and effective agents for treating aortic valve calcification is urgently needed. It has been recognized that osteogenic differentiation of aortic valve interstitial cells (AVICs) is the fundamental hallmark of CAVD [5, 6]. Therefore, an effective therapeutic strategy for CAVD may be prevent the phenotypic switching of AVICs to an osteoblast-like phenotype.

Protein tyrosine phosphorylation is an essential mechanism for numerous biological processes, including immunity, proliferation, differentiation, and calcification [7]. Aberrant protein tyrosine phosphatases (PTPs) may cause several human diseases, such as cardiovascular diseases, diabetes, and autoimmune disorders [8, 9]. The complement of PTPs includes the classic, phosphotyrosine (pTyr)-specific PTP and the VH1-like or dual specificity protein phosphatase (DUSP) subfamilies [10]. In particular, PTPN1, also known as protein tyrosine phosphatase nonreceptor type 1, has been associated with several diseases, especially those that affect the metabolic and cardiovascular systems. Dawn Thompson et al. showed that PTPN1 ablation attenuated the formation of atherosclerotic plaques in an LDLR-/- mouse model [11]. Targeted inhibition of PTPN1 is known to protect against cardiovascular dysfunction and mortality [12]. Additionally, Chiu-Fen Yang et al. found that PTPN12 levels were increased in the myocardium during ischaemia/reperfusion, and PTPN12 activation was harmful in ischaemia/reperfusion [13]. After treatment with auranofin, an inhibitor of PTPN12, the infarct size was significantly reduced and cardiac function was improved in a mouse model of ischaemia/reperfusion, suggesting that PTPN12 contributes to myocardial ischaemia/reperfusion injury [13]. A study showed that DUSP26, a member of the PTP family, was expressed at significantly higher levels in the calcific aortic valve, and ablation of DUSP26 ameliorated aortic valve calcification in human AVICs and in ApoE^-/-^ mice fed a high cholesterol diet [14]. Indeed, PTPs are involved in cardiovascular diseases, including aortic valve calcification.

Protein tyrosine phosphatase nonreceptor type 22 (PTPN22), which is an intracellular nonreceptor-like PTP, dephosphorylates threonine and tyrosine residues in proteins [15]. PTPN22 has been linked to several chronic inflammatory disorders, such as systemic lupus erythematosus, rheumatoid arthritis, Crohn’s disease, and diabetes [16]. It has been established that PTPN22 regulates T-cell receptor signalling [17]. Furthermore, Won Jin Ho et al. found that inhibition of PTPN22 protected against tumour growth [18]. Recent studies have shown that PTPN22 ablation reduces tail-bleeding time and accelerates arterial thrombus formation, suggesting that PTPN22 is a novel potential target for thrombotic or cardiovascular diseases [19]. Furthermore, several lines of evidence suggest that PTPN22 is implicated in atherosclerosis [20, 21], a disease that shares common risk factors and pathogenesis with CAVD. Hence, the potential effect of PTPN22 on CAVD remains to be investigated using comprehensive approaches.

In the present study, we examined the role of PTPN22 in CAVD by utilizing genetic and pharmacologic approaches in vitro and in vivo. More importantly, we discovered a new PTPN22 inhibitor, 13-hydroxypiericidin A 10-O-α-D-glucose (1 → 6)-β-D-glucoside (S18), which is a marine-derived product. The results of our study reveal that PTPN22 is crucial to the pathogenesis of CAVD, and S18, a novel PTPN22 inhibitor, is identified as a potential therapeutic drug.

## Methods

### Collection of aortic valve leaflets from patients

In 2021–2022, we collected calcified aortic valves from six patients with CAVD who had undergone valve replacement (Table S1). Noncalcified aortic valves were obtained intraoperatively from six patients undergoing aortic valve replacement after acute aortic dissection. Those with infective endocarditis, congenital valve disease, and rheumatic heart disease were excluded. The diagnosis of CAVD was made when the leaflet thickness was greater than 3 mm, the peak AV velocity was greater than 1.5 m/s, and the echogenicity of the aortic valve was increased compared to that of the aortic root as a control [22]. All patients involved in this study provided written informed consent. During the course of this study, the protocol was performed in compliance with the Declaration of Helsinki and was approved by Nanfang Hospital, Southern Medical University (ID: NFEC-2021-064).

### Cell culture and treatment

Human AVICs were isolated and cultured as previously reported [23]. Briefly, after the collagenase digestion of aortic valve leaflets, cells were collected by centrifugation. We cultured AVICs in M199 growth medium (Gibco, C11150500BT, USA), which contains 100 units of penicillin G, 100 μg of streptomycin, and 10% foetal bovine serum. Following passage 3, cells reached 80%-90% confluence and were then used in subsequent experiments. For osteogenic differentiation, cells were cultured with osteogenic medium (OM) (growth medium supplemented with 10.0 mmol/L β-glycerophosphate, 10.0 nmol/L dexamethasone, 4.0 μg/ml cholecalciferol and 8.0 mmol/L CaCl_2_) [24]. For some experiments, cells were transfected with a control vector (pCMV3-control, SinoBiological, Beijing, CN), PTPN22 expression vector (pCMV3-PTPN22, SinoBiological, Beijing, CN), or PTPN22-specific small interfering RNA (siRNA, OBIO Biotechnology, Shanghai, CN) by Lipofectamine 3000 (ThermoFisher, L3000015, USA) according to the manufacturer’s protocol. Scramble RNA was employed as negative control. Human AVICs were then treated with S18 as indicated.

### Experimental mouse model

Animal experiments were conducted in accordance with the guidelines of Directive 2010/63/EU of the European Parliament on the protection of animals used for scientific purposes and the ARRIVE guidelines. This study was approved by the Ethics Committee for Animal Experiments of Southern Medical University (ID: NFYY-2021-1159).

Male apolipoprotein E-deficient (ApoE^-/-^, 8–10 weeks) mice were acquired from the Guandong Medical Lab Animal Cline Centre (Guangzhou, China) and housed in a pathogen-free, temperature-controlled environment under a 12-h light/dark cycle. Mice were fed a high-fat diet (HFD: 21% fat, 0.15% cholesterol) for 24 weeks to develop CAVD [25]. All diets were purchased from Guangdong Medical Lab Animal Centre. We employed Vevo 2100 ultrasound (Visual Sonics, Toronto, ON, Canada) after anaesthesia with 2% isoflurane to detect the morphology of the aortic valve and the function of the heart. Finally, the mice were euthanized with an intraperitoneal injection of sodium pentobarbital (50 mg/kg), and hearts were collected.

8–10 week-old male C57BL/6 mice were obtained from the Laboratory Animal Center of Southern Medical University and housed in a pathogen-free, temperature-controlled environment under a 12-h light/dark cycle. The health status of every animal was monitored twice weekly, such as weight, food intake, and physical activity. We employed an aortic valve injury (AVI) model, which was established as previously described [26, 27]. Briefly, anaesthetized mice had their chests shaved to expose the right carotid artery. In the right carotid artery, a spring wire was used for angioplasty and then inserted into the left ventricle. The aortic valve leaflets were scratched by the body of the wire 30 times. Mice in the sham control group underwent the same procedure without wire insertion into the left ventricle. After 12 weeks, we employed Vevo 2100 ultrasound (Visual Sonics, Toronto, ON, Canada) after anaesthesia with 2% isoflurane to detect the morphology of the aortic valve and the function of the heart. Finally, the mice were euthanized, and hearts were collected.

To determine the role of PTPN22 in vivo, we employed PTPN22^−/−^ mice (C57BL/6) generated by Cyagen Biosciences Inc. (stock no. KOCMP-71198-Otud1-B6J-VA, Cyagen Bioscience, Jiangsu, CN) according to a previous report [19]. A total of 15 male mice were randomly assigned to 3 groups: sham control (*n* = 5), AVI (*n* = 5), and AVI + PTPN22^−/−^ (*n* = 5). A case number estimation using data from a prior study [28] produced a group size of *n* = 5. To confirm the efficacy of S18 in vivo, a total of 15 male mice were randomly assigned to 1 of 3 groups: sham control (*n* = 5), AVI + vehicle control (*n* = 5), and AVI + S18 (1 mg/kg, *n* = 5). S18 was administered daily by i.p. injection at a dose of 1 mg/kg based on pilot studies [29]. The experimental protocol is shown in supporting information Fig. S9 in detail.

### Statistical analysis

Data are expressed as the means ± SEM. Statistical analyses were performed using GraphPad Prism 7.0 (GraphPad, CA, USA). First, to verify whether the data obey a normal distribution, a normality test and F test were used. If the data showed a normal distribution, an unpaired t test was performed to compare the two groups. Comparisons between multiple groups were performed using one-way analysis of variance (ANOVA) combined with a Tukey–Kramer test or Dunnett post hoc test. A *P* value less than 0.05 was considered statistically significant.

## Results

### Higher levels of PTPN22 are present in aortic valves from CAVD patients and in different mouse models of CAVD

**Fig. 1:**
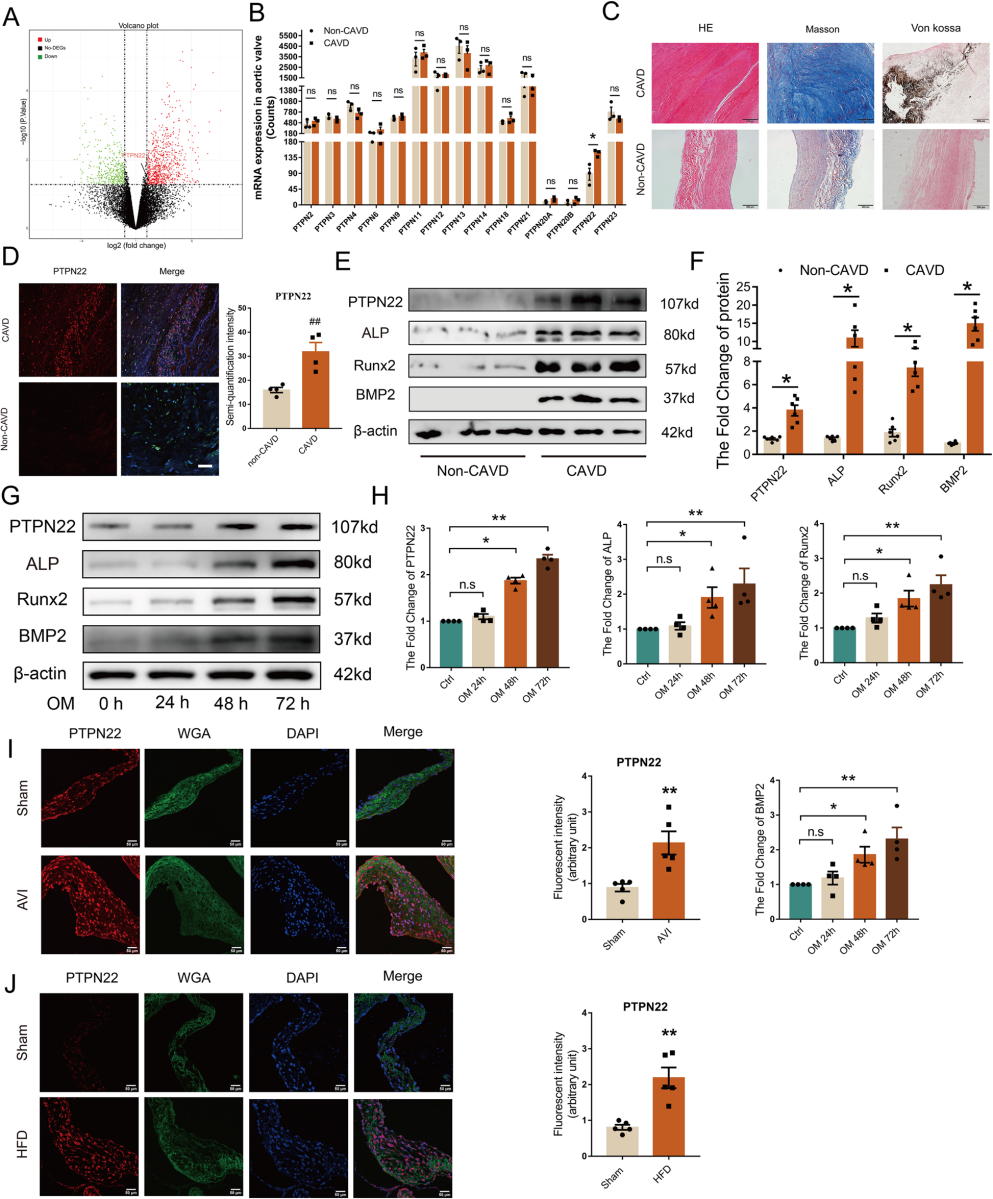
PTPN22 expression is increased in aortic valves from CAVD patients. The aortic valves were collected from CAVD and non-CAVD patients. **A**-**B** Volcano plot and transcriptome sequencing analysis of the non-CAVD and CAVD groups indicated that PTPN22 expression is upregulated in tissue from CAVD patients. *n* = 3. **C** H&E, Masson’s trichrome, and Von Kossa staining for calcified valve leaflets. Scale bar = 200 μM. **D** Immunofluorescent staining of PTPN22 (red) in tissue (DAPI: blue, vimentin: green). *n* = 4. Scale bar = 100 μM. **E**-**F** The protein expression of PTPN22 in tissue was detected by immunoblotting. *n* = 6. **G**-**H** Human AVICs were stimulated with OM for different time periods. PTPN22 protein expression in human AVICs was detected by immunoblotting. *n* = 4. **I**-**J** Immunofluorescent staining was used to detect PTPN22 (red) expression in different mouse models of CAVD (DAPI: blue, WGA: green). *n* = 5. Scale bar = 50 μM. All data are shown as means ± SEM. *p < 0.05; **p < 0.01

To detect PTPs candidates in the pathogenesis of CAVD, we first analysed the transcriptome sequencing of three human calcific aortic valves and three normal aortic valves. The transcriptome sequencing results showed that PTPN22 was upregulated 1.8-fold in calcific aortic valves (|fold change (FC)|≥ 1.5 and *p* ≤ 0.05), suggesting that PTPN22 may play a crucial role in the progression of CAVD (Fig. 1A-B). Thus, we focused on PTPN22 in our subsequent experiments. Next, we collected aortic valves from CAVD patients and non-CAVD patients and performed HE, Masson’s trichrome, and Von Kossa staining (Fig. 1C). Immunofluorescence staining showed that the levels of PTPN22 in the calcific valves were higher than those in the non-CAVD group (Fig. 1D, ##*p* < 0.01). Similar results were obtained by western blot (Fig. 1E-F, *p < 0.05; **p < 0.01) and qPCR (Additional file 1: Fig. S1A) analysis of the aortic valve. Moreover, we exposed human AVICs to OM for different time periods and found that the level of PTPN22 was upregulated in a time-dependent manner, suggesting the potential role of OM in mediating PTPN22 induction in human AVICs (Fig. 1G-H, *p < 0.05; **p < 0.01). Furthermore, we investigated the role of PTPN22 in a mouse model induced by direct wire injury. Figure 1I shows that the PTPN22 protein levels were upregulated in the mouse model of CAVD, whereas hardly detected in the sham control mice (*p < 0.05; **p < 0.01). We also employed ApoE^−/−^ mice treated with a high-fat diet (HFD) to develop a CAVD model [25]. Similar results were obtained when PTPN22 levels were measured in ApoE^−/−^ mice (Fig. 1J, *p < 0.05; **p < 0.01). These data provide direct evidence that PTPN22 is dysregulated in the progression of CAVD.

### PTPN22 promotes osteoblast-like differentiation in human AVICs

**Fig. 2:**
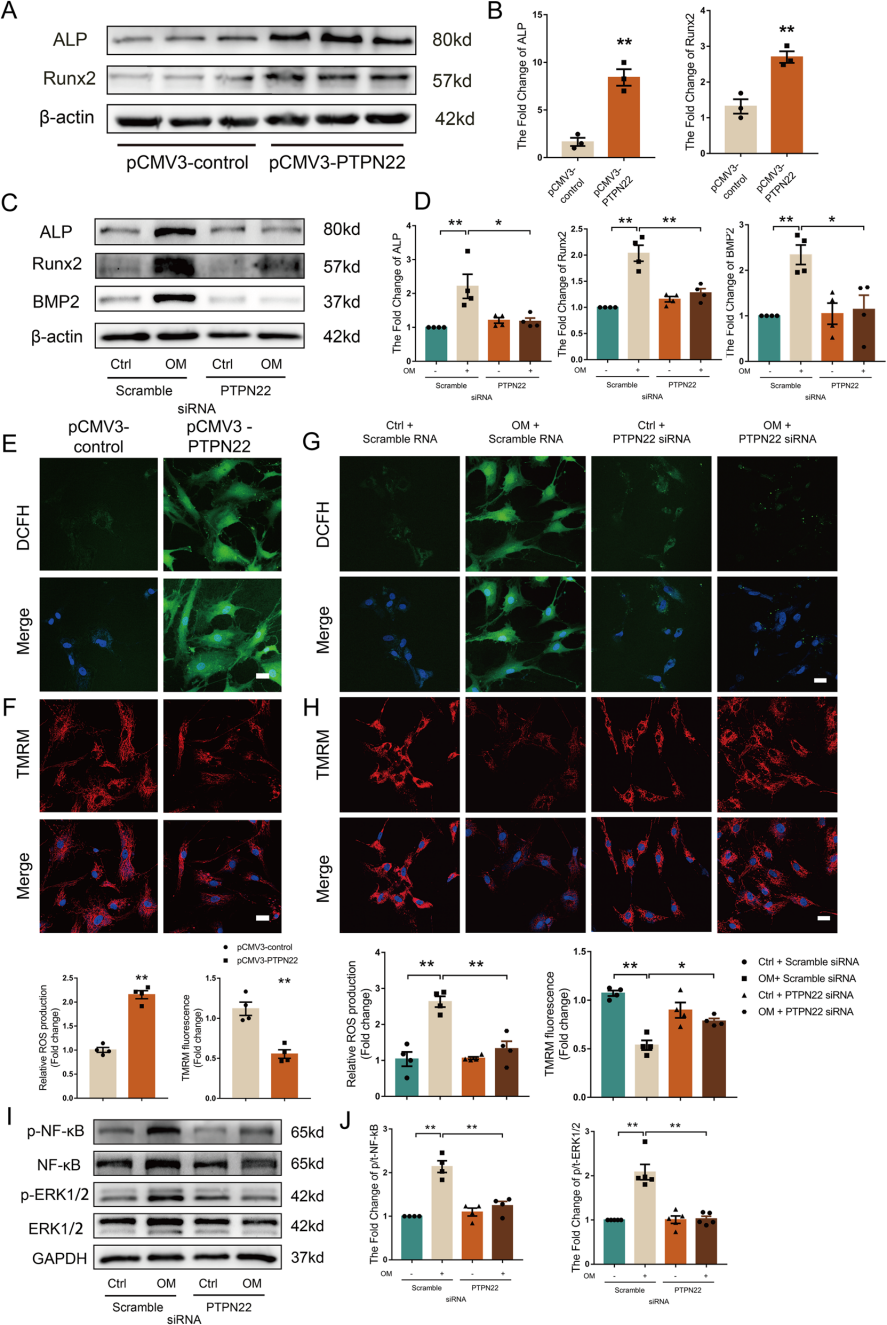
PTPN22 mediates OM-induced osteogenic responses in vitro. **A**-**B** AVICs were transfected with the PTPN22 expression vector (pCMV3-PTPN22) or empty vector ((pCMV3-control). The immunoblotting results show the expression of ALP and Runx2 in vitro. *n* = 3. **C**-**D** Cells were treated with PTPN22 siRNA following stimulation with OM. Representative western blot and quantitative data on the relative abundance of ALP, Runx2 and BMP2 in the different groups. Cells were transfected with pCMV3-PTPN22. Images of DCFH-DA staining **E** and TMRM staining (**F**). *n* = 4. Scale bar = 25 μm. AVICs were transfected with PTPN22 siRNA. Images of DCFH-DA staining (**G**) and TMRM staining (**H**). *n* = 4. Scale bar = 25 μm. **I**-**J** Western blot analysis shows the levels of NF-κB and ERK1/2 phosphorylation after PTPN22 knockdown. *n* = 4. All data are shown as the means ± SEM. *p < 0.05; **p < 0.01

To examine the functional effects of PTPN22 on CAVD, we employed the gain-of-function approach by forced PTPN22 protein expression in human AVICs. To this end, human AVICs were transfected with the PTPN22 expression vector (pCMV3-PTPN22) or empty vector (pCMV3-control). The results revealed that the expression of alkaline phosphatase (ALP), and runt-related transcription factor-2 (Runx2) was induced in human AVICs transfected with pCMV3-PTPN22 (Fig. 2A-B, **p < 0.01), suggesting a pro-osteogenic action of PTPN22 in human AVICs. Additionally, we also employed a small interfering RNA (siRNA) for silencing PTPN22 expression. Notably, PTPN22 knockdown significantly inhibited the expression of ALP, Runx2 and bone morphogenetic protein-2 (BMP2) (Fig. 2C-D, *p < 0.05; **p < 0.01), suggesting that PTPN22 mediates aortic valve calcification.

Given that mitochondrial stress promotes the osteogenic differentiation of AVICs and calcium deposition in valvular tissue [30]. We next investigated the functional role of PTPN22 in mitochondrial stress. We found that ROS expression was induced and the mitochondrial membrane potential (MMP) was reduced in cells treated with pCMV3-PTPN22 (Fig. 2E-F, *p < 0.05; **p < 0.01). Conversely, knockdown of PTPN22 through siRNA in human AVICs reduced the levels of ROS and regulated MMP (Fig. 2G-H, *p < 0.05; **p < 0.01), suggesting that PTPN22 plays a critical role in mediating mitochondrial stress in human AVICs.

Several lines of evidence have documented that the NF-κB and ERK1/2 signalling pathways mediate vascular calcification and osteogenic differentiation [31–33]. In addition, KEGG pathway analysis of our transcriptome sequencing data suggested that genes in the NF-κB and MAPK/ERK signalling pathways were enriched in the calcific aortic valve (Additional file 1: Fig. S1B). We then examined the role of PTPN22 in modulating the NF-κB and ERK1/2 signalling pathways.

Genes from CAVD samples in transcriptome sequencing data were ranked by their relative PTPN22 expression, including the top 10% (PTPN22^Hi^) and the bottom 10% (PTPN22^Low^), by gene set enrichment analysis (GSEA). The results revealed that PTPN22^Hi^ aortic valve samples were enriched in the expression of gene signatures related to the NF-κB signalling pathway compared with that in the PTPN22^Low^ samples, suggesting that PTPN22 is positively correlated with the NF-κB signalling pathway (Additional file 1: Fig. S1C). Western blot analysis revealed that siRNA-mediated PTPN22 inhibition significantly decreased the levels of NF-κB and ERK1/2 phosphorylation (Fig. 2I-J, **p < 0.01), suggesting the essential role of PTPN22 in mediating NF-κB and ERK1/2 signalling in the osteogenic differentiation of human AVICs.

### Mice with PTPN22 deficiency are protected against CAVD

**Fig. 3:**
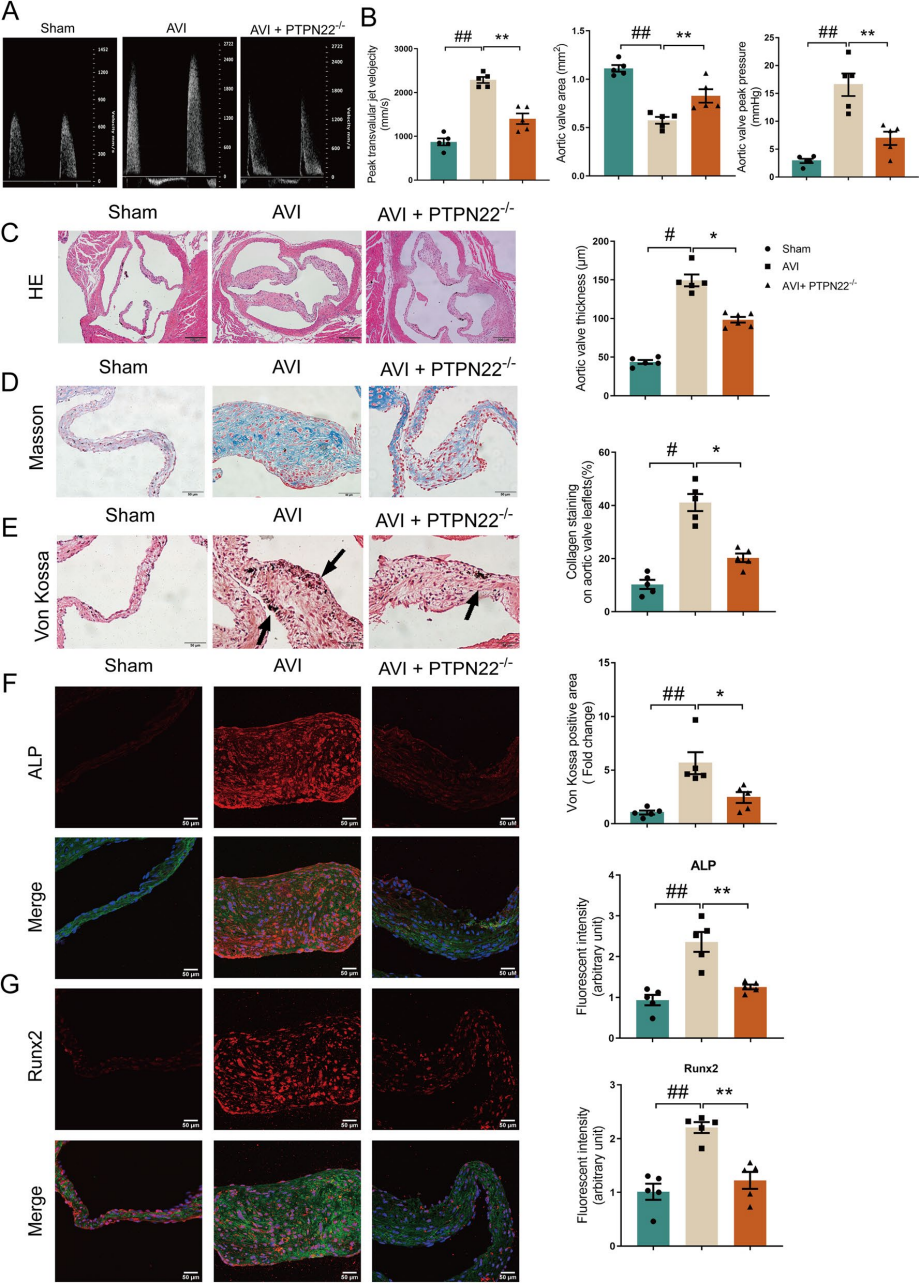
PTPN22 deletion mitigates aortic valve lesions in vivo. Echocardiographic data in a wire injury-induced CAVD mouse model. **A**-**B** Peak transvalvular jet velocity, aortic valve peak pressure, and AVA. **C** Image of aortic valves in the aortic valve calcification model. H&E staining of aortic valves after wire injury. Scale bar = 200 μm. **D** Masson’s trichrome staining. Scale bar = 50 μm. **E** Von Kossa staining. Scale bar = 50 μm. **F**-**G** Immunofluorescence images of the aortic valve (DAPI: blue, WGA: green). Scale bar = 50 μm. *n* = 5. Values are the means ± SEMs. #*p* < 0.05, ##*p* < 0.01 vs. sham group; *p < 0.05, **p < 0.01 vs. AVI + PTPN22^−/− ^^group^

We next examined the effect of PTPN22 on aortic valve lesions in vivo, and PTPN22^−/−^ mice were utilized in this study. At 3 months after the wire injury procedure, an ultrasonic cardiogram was performed. The mice undergoing aortic valve injury (AVI) showed a dramatic increase in peak transvalvular jet velocity and aortic valve peak pressure and a decrease in aortic valve area (AVA, Fig. 3A-B, ##*p* < 0.01, **p < 0.01), and PTPN22 knockdown restored these changes. We then examined the morphology, collagen and calcification of the valve leaflets in these mice. Figure 3C shows that the thickness of aortic valve leaflets was markedly decreased in PTPN22^−/−^ mice. Moreover, ablation of PTPN22 normalized the level of collagen and calcification in mice (Fig. 3D-E, #*p* < 0.05, *p < 0.05, Additional file 1: Fig. S7). Consistently, immunofluorescence analysis indicated that ablation of PTPN22 suppressed the expression of ALP and Runx2 (Fig. 3F-G, ##*p* < 0.01, *p < 0.05, **p < 0.01). These observations further suggest a significant effect of PTPN22 in promoting aortic valve lesions in a wire injury-induced CAVD mouse model.

### Piericidin diglycoside S18 is a novel inhibitor of PTPN22

**Fig. 4:**
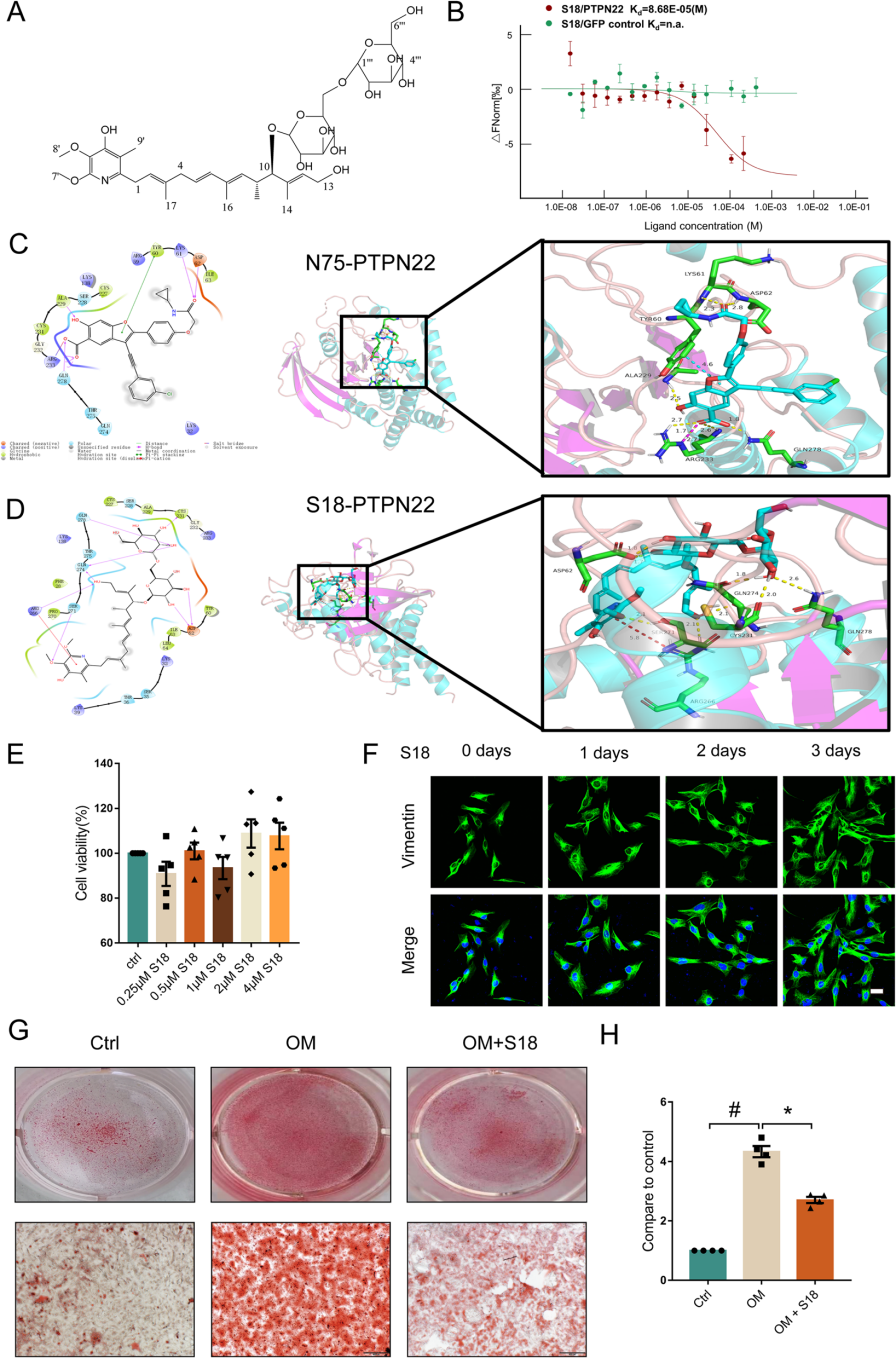
Discovery and characterization of compound S18, a novel inhibitor of PTPN22. **A** The molecular structure of S18. **B** The binding affinities of S18/PTPN22 (K_d_ = 86.8 μM) and S18/GFP control (K_d_ = no binding) were detected by MST assay. Values are the means ± SEM. *n* = 3. *p < 0.05. The detailed docking mode of (**C**) N75/PTPN22 and (**D**) S18/PTPN22 with docking pock. **E** Cell viability of human AVICs treated with S18 for 3 days. *n* = 5. **F** Cell morphology of human AVICs treated with S18 for 3 days. Scale bar = 25 μm. **G**-**H** Alizarin Red S staining of cells cultured under different conditions. *n* = 4. Values are the means ± SEM. #*p* < 0.05 vs. the control group, *p < 0.05 vs. the OM group

After establishing the potential role of PTPN22 in CAVD, we then sought to discover a novel inhibitor of the PTPN22 protein to alleviate valve calcification. Piericidin diglycoside S18 was discovered from an actinomycete strain, Streptomyces pasmmoticus SCSIO NS126, which is derived from a mangrove sediment sample in the Pearl River estuary of the South China Sea. As previously reported [34], S18, a specific piericidin diglycoside, was identified as 13-hydroxypiericidin A 10-O-α-D-glucose (1 → 6)-β-D-glucoside through HRMS and NMR analyses (Fig. 4A). We previously found that piericidin diglycoside S18 suppresses inflammation in human AVICs. Therefore, we hypothesized that S18 inactivates PTPN22. Strikingly, the results of microscale thermophoresis (MST) analysis revealed that S18 had a strong binding affinity to PTPN22 with a K_d_ value of 86.8 μM (Fig. 4B). Concomitantly, molecular docking analysis was performed to examine the interaction between S18 and PTPN22. A homology model of PTPN22 (PDB code: 4J51) was selected, and then silico molecular docking analysis was performed. N75 was selected as the positive control [35]. The detailed 2-dimensional (2D) and 3D interactions between S18, N75 and PTPN22 are shown in Fig. 4C and D, with docking scores of -6.672 and -4.732, respectively. Moreover, the glycoside group in S18 formed a strong hydrogen bond interaction with the active site residues ASP26, GLN274, and GLN278. The pyridinol group interacted with the active site residues ARG266 and SER274. Overall, these findings reveal a strong binding affinity of S18 with the PTPN22 protein.

To assess the toxic effects of S18 on human AVICs, we employed a CCK8 assay to assess cell viability. Figure 4E shows no difference in cell viability when S18 was present at concentrations below 4 μM. Similarly, immunofluorescent staining indicated that the cell morphology of human AVICs had no marked change when treated with S18 (0.5 μM) for 3 days (Fig. 4F). Then, we employed Alizarin Red S staining to test the pharmacological efficacy of S18. Interestingly, the results revealed that 0.5 µM S18 treatment decreased the accumulation of calcium deposits in human AVICs cultured with OM for 14 days (Fig. 4G-H, #*p* < 0.05, *p < 0.05). These data suggest that S18 is potential to inhibit osteogenic responses in vitro.

### Piericidin diglycoside S18 inhibits OM-mediated osteogenic responses in AVICs

**Fig. 5:**
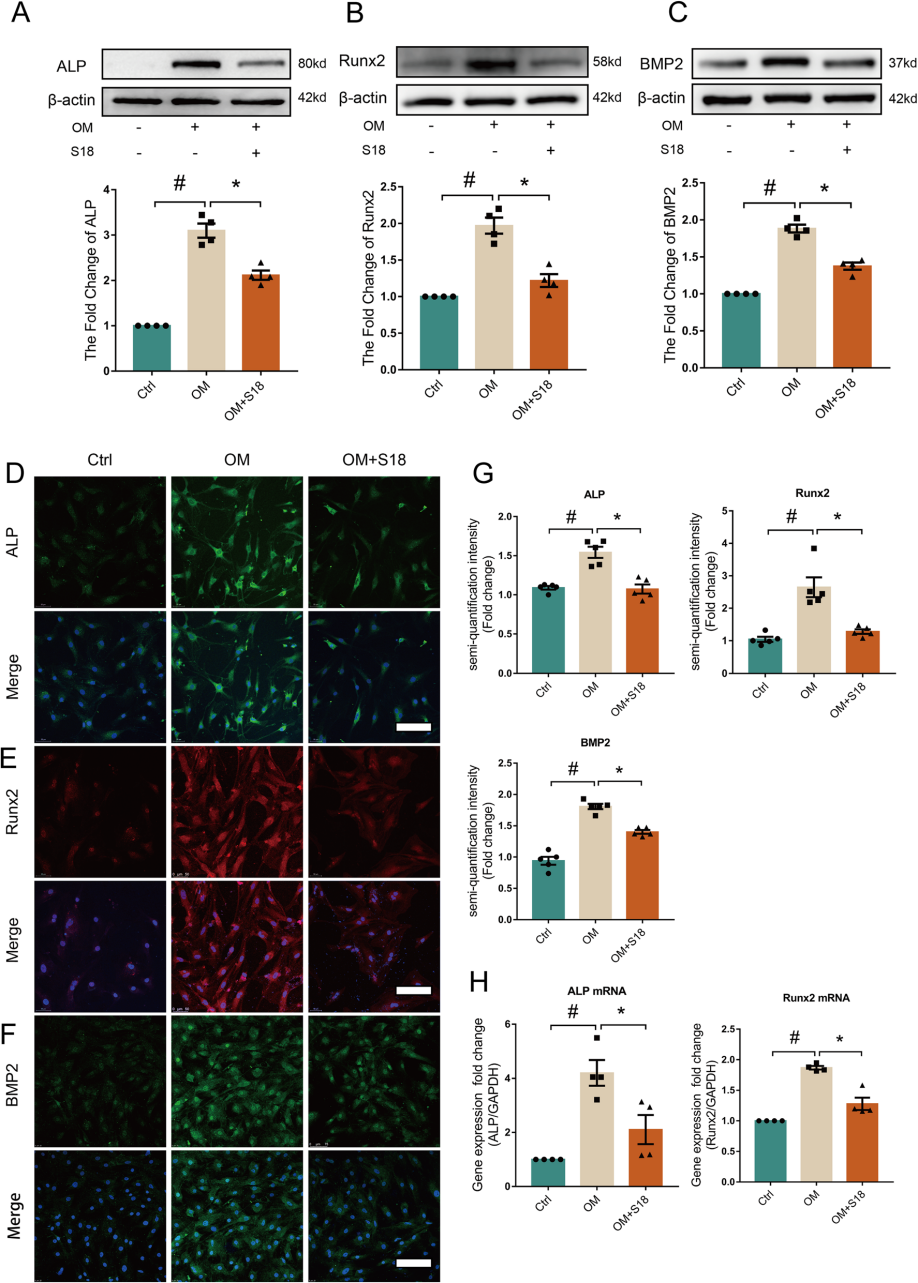
Piericidin diglycoside S18 exhibited a remarkable anti-osteogenic responses effect. Human AVICs were cultured with OM and treated with or without 0.5 µM S18 for 3 days. **A**-**C** Western blotting and quantification analysis showed the expression of ALP, Runx2 and BMP2 in human AVICs. *n* = 4. **D**-**G** Immunofluorescence staining of ALP (green), Runx2 (red) and BMP2 (green) in human AVICs (DAPI: blue). *n* = 5. Scale bar = 100 μm. **H** qRT-PCR was employed to measure ALP mRNA and Runx2 mRNA expression. *n* = 4. Values are the means ± SEM. #*p* < 0.05 vs. the control group, *p < 0.05 vs. the OM group

To determine the therapeutic efficacy of S18, AVICs were cultured in OM containing S18 for 3 days. We analysed the expression of ALP, Runx2 and BMP2. Immunoblotting (Fig. 5A-C, #*p* < 0.05, *p < 0.05) and immunofluorescence staining (Fig. 5D-G, #*p* < 0.05, *p < 0.05) showed that the levels of osteogenic responses-related genes were decreased by S18 under OM conditions. Additionally, following treatment with S18 in OM conditions for 2 days, the levels of ALP mRNA and Runx2 mRNA were decreased compared with those cultured in OM alone (Fig. 5H, #*p* < 0.05, *p < 0.05). Therefore, these data provide strong evidence that S18 negatively regulates osteogenic responses in AVICs.

### Piericidin diglycoside S18 suppresses PTPN22-mediated osteogenic responses in vitro

**Fig. 6:**
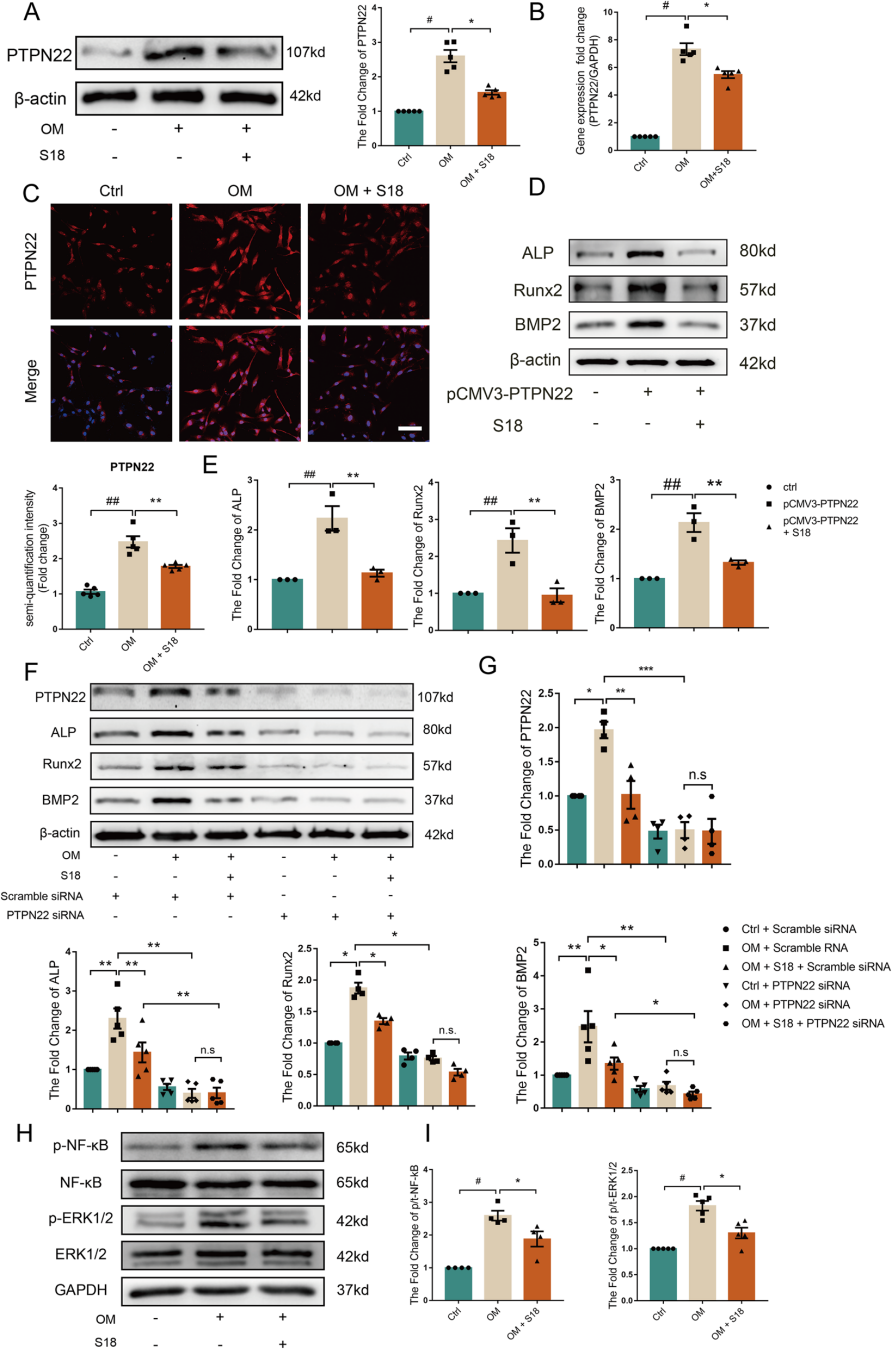
Piericidin diglycoside S18 suppresses PTPN22-mediated osteogenic responses in vitro. Human AVICs were pretreated with S18 for 1 h followed by exposure to OM for 3 days. **A**-**B** The PTPN22 protein and mRNA levels were decreased in human AVICs treated with S18. *n* = 5. **C** Representative micrographs of the immunofluorescence staining of PTPN22 (red) expression in human AVICs treated with S18 (DAPI: blue). *n* = 5. Scale bar = 100 μm. Values are the means ± SEM. #*p* < 0.05, ##*p* < 0.01 vs. the control group, *p < 0.05; **p < 0.01 vs. the OM group. **D**-**E** Human AVICs were transfected with pCMV3-PTPN22 or pCMV3-control. Immunoblotting results show ALP, Runx2 and BMP2 expression in human AVICs. *n* = 3. Values are the means ± SEM. ##*p* < 0.01 vs. control group, **p < 0.01 vs. pCMV3-PTPN22 group. **F**-**G** AVICs were treated with nonspecific control or PTPN22-specific siRNA and treated with OM and S18 for the indicated times. Representative immunoblots and corresponding quantifications show that ALP, Runx2 and BMP2 expression was decreased in PTPN22 knockdown human AVICs. *n* = 5. Values are the means ± SEM. *p < 0.05, **p < 0.01, n.s. **H**-**I** Western blot analysis was used to detect the levels of NF-κB and ERK1/2 phosphorylation. *n* = 4. Values are the means ± SEM. #*p* < 0.05 vs. control, *p < 0.05 vs. the OM + S18 group

Next, we assessed the role of S18 in PTPN22-mediated osteogenic responses. As expected, treatment with S18 markedly reduced OM-induced protein and mRNA levels of PTPN22 in cultured human AVICs (Fig. 6A-B, #*p* < 0.05, *p < 0.05), underscoring the potent ability of S18 to abolish OM-triggered PTPN22 expression. Similarly, immunofluorescence staining revealed that S18 suppressed the expression of PTPN22 in cells treated with OM (Fig. 6C, ##*p* < 0.01, **p < 0.01). Furthermore, S18 obliterated the ALP, Runx2 and BMP2 expression induced by PTPN22 overexpression alone (Fig. 6D-E, ##*p* < 0.01, **p < 0.01). Subsequently, we also employed siRNA to knock down PTPN22 expression. Interestingly, the pharmacological effect of S18 was abolished by PTPN22 silencing (Fig. 6F-G, *p < 0.05, **p < 0.01). These findings identified that piericidin diglycoside S18 is a potent inhibitor of PTPN22 to repress osteogenic responses in human AVICs.

We next determined the signalling mechanism by which S18 prevents calcification in human AVICs. Accordingly, western blot analysis showed that the levels of NF-κB and ERK1/2 phosphorylation were decreased by 70% in S18-treated cells compared to cells cultured in OM alone (Fig. 6H-I, #*p* < 0.05, *p < 0.05). In addition, immunofluorescence staining revealed that S18 inhibited the nuclear translocation of NF-κB p65 (Additional file 1: Fig. S2A). These data provide evidence that piericidin diglycoside S18 inhibits calcification in human AVICs via the NF-κB and ERK1/2 signalling pathways.

Mitochondrial stress exerts crucial effects on the development of cardiovascular disease [30, 36, 37]. We next tested the role of S18 in mitochondrial stress signals, and the results showed that ROS levels were increased in OM-treated human AVICs (Additional file 1: Fig. S3A). Conversely, fluorescence images of ROS showed that S18 supplementation markedly decreased ROS levels (Additional file 1: Fig. S3A). Additionally, we detected MMP in human AVICs by TMRM staining. The results showed that S18 administration regulates the MMP in human AVICs (Additional file 1: Fig. S3B). These findings suggest that S18 modulates OM-induced mitochondrial dysfunction in human AVICs.

To further investigate the effect of PTPN22 on mitochondrial stress, human AVICs were treated with pCMV3-PTPN22 or pCMV3-control prior to treatment with S18 for 3 days. As shown in Additional file 1: Fig. S3C-D, the overexpression of PTPN22 induced ROS production and decreased MMP in human AVICs. In contrast, after treatment with S18, mitochondrial stress was significantly restored. Thus, these results suggest that S18 prevents against mitochondrial stress by modulating PTPN22.

### Piericidin diglycoside S18 alleviates aortic valve lesions in the AVI mouse model

**Fig. 7:**
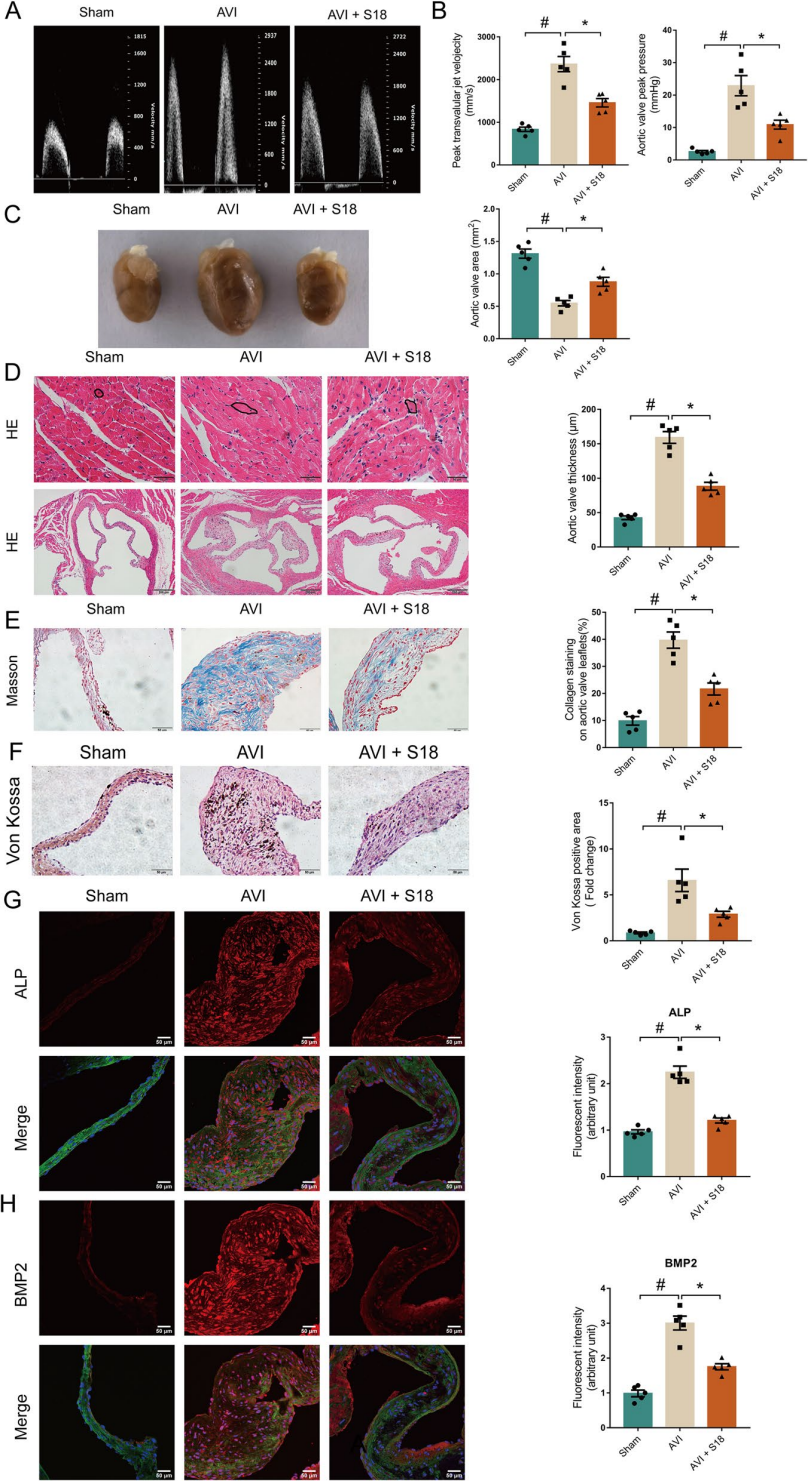
Piericidin diglycoside S18 protects the aortic valve against injury in an established CAVD model. Echocardiographic data in wire injury-induced CAVD moues model. **A**-**B** Peak transvalvular jet velocity, aortic valve peak pressure, and AVA. **C**-**D** Images of aortic valves in the aortic valve calcification model. H&E staining of aortic valves after wire injury. Scale bar = 200 μm. **E** Masson’s trichrome staining. Scale bar = 50 μm, **F** Von Kossa staining. Scale bar = 50 μm. **G**-**H** Immunofluorescence images of the aortic valve (DAPI: blue, WGA: green). Scale bar = 50 μm. *n* = 5. All data are the means ± SEM. #*p* < 0.05, vs. the sham group, *p < 0.05 vs. the AVI and S18 group

To further test the therapeutic efficacy of S18 on CAVD in vivo, we employed an AVI model in this study [27, 38]. Wild-type mice underdoing subjected to AVI were treated by intraperitoneal injection of S18 (1 mg/(kg*d)) for 2 months [34]. Figure 7A-B shows that S18 markedly decreased aortic velocity, peak transvalvular jet velocity, and aortic valve peak pressure and increased AVA in mice subjected to wire injury. Moreover, the cross-sectional areas of cardiomyocytes and the thickness of aortic valve leaflets were rapidly increased in the AVI group while were decreased by S18 administration (Fig. 7D, #*p* < 0.05, *p < 0.05). Masson staining illustrated that S18 reduced fibrosis in the aortic valve (Fig. 7E, #*p* < 0.05, *p < 0.05). Correspondingly, Von Kossa staining and Alizarin red S staining revealed that the calcium deposition in the aortic valve was mitigated by S18 therapy after wire injury (Fig. 7F, Additional file 1: Fig. S8).

Additionally, we found that the levels of Runx2, ALP, and BMP2 were upregulated in the AVI model group, while S18 partially moderated these changes in valve tissues (Fig. 7G-H, Fig. S4A, #*p* < 0.05, *p < 0.05). Furthermore, PTPN22 expression was increased in the AVI group. S18 treatment suppressed the expression of PTPN22 in the AVI group (Additional file 1: Fig. S4D). These data indicate the anti-inflammatory and anti-calcification effects of S18 in vivo.

## Discussion

The major novel findings in the present study are as follows: (1) PTPN22 expression is increased in aortic valve from CAVD patients and in human AVICs stimulated with OM; (2) PTPN22 plays a vital role in mitochondrial stress, and PTPN22 silencing alleviates mitochondrial dysfunction and osteogenic responses in vitro as well as aortic valve calcification in vivo; (3) piericidin diglycoside S18, a novel PTPN22 inhibitor, was discovered; and (4) pharmacological piericidin diglycoside S18 significantly attenuates aortic valve calcification in animal experiments and in vitro studies. On the basic of our knowledge, our study provides direct evidence for a novel target for therapeutic intervention in CAVD and demonstrates the anti-CAVD properties of piericidin diglycoside S18.

Increasing evidence indicates that PTPs exert great effects on the progression of cardiovascular diseases. Dong N et al. found that DUSP26 expression is related to the severity of CAVD and is increased in calcified aortic valves and in ApoE^−/−^ mice. DUSP26 ablation markedly reduces osteogenic markers in vitro and mitigates aortic valve calcification in ApoE^−/−^ mice [14]. Some studies show that PTPN22 is associated with chronic inflammatory diseases, and PTPN22 knockdown reduces IL-1β secretion [18]. PTPN22 knockdown reduced the levels of inflammatory factors, including ICAM-1, IL-8 and MCP-1, in THP1 monocytes stimulated with IFN-γ [39, 40]. Furthermore, PTPN22 interacts with TRAF3, promotes TRAF3 polyubiquitination, and then activates the serine-threonine kinases TBK1 and IKK. Subsequently, the transcription factors IRF3 and IRF7, which are substrates for TBK1 and IKK, translocate into the nucleus and activate type I interferon transcription [15, 41, 42]. In this regard, PTPN22 is likely involved in the pathogenesis of CAVD. Our study is the first to report that PTPN22 expression is increased in aortic valve tissue from CAVD patients, ApoE^−/−^ mice fed with HFD, and a wire injury-induced CAVD mouse model, which is in line with the effects of DUSP26. Moreover, PTPN22 expression was induced in human AVICs treated with OM. These results indicate that the upregulation of PTPN22 is a molecular pathogenic feature in calcific aortic valves.

In view of PTPN22 induction with OM, it is important to elucidate the potential effect of PTPN22 activation on valvular cells. We showed that PTPN22 overexpression was sufficient to induce osteogenic marker expression in human AVICs. Consistent with this notion, siRNA-mediated knockdown or S18-mediated inhibition dramatically suppresses OM-induced osteogenic responses in cells. These observations indicate a strong pro-calcific action of PTPN22. Moreover, PTPN22 ablation protected mice against the progression of CAVD after AVI, providing strong evidence for PTPN22-mediated aortic valve calcification. These results are also consistent with the effect of DUSP26 and in harmony with observations in vitro. Furthermore, similar to the genetic approach, the PTPN22 inhibitor S18 suppressed ALP, Runx2 and BMP2 levels in human AVICs and alleviated calcification in wire injury-induced aortic valves. Taken together, the results of our study on the role of PTPN22 in CAVD are quite cohesive, as shown by in vivo and in vitro data, as well as genetic and pharmacologic techniques.

The mitochondria provide the majority of energy in cells and maintain myocardial excitation–contraction coupling [37]. Mitochondrial dysfunction and alterations in mitochondrial morphology result in abnormal ROS production, causing mitochondrial stress and inflammatory responses [43]. An overt increase in ROS was observed in ApoE^−/−^ mice fed with HFD. In cardiac tissue, excessive ROS promote postischemic inflammatory infiltration, leading to cardiac hypertrophy, fibrosis, and necrosis [23, 44]. Additionally, it has been demonstrated in animal and in vitro models that mitochondrial stress is associated with the progression of CAVD [30, 45]. Oxidative stress, especially ROS, facilitates lipid infiltration and inflammation, which induce calcification and promote the progression of CAVD [46]. Moreover, ROS also induce BMP2 expression, which then modulates osteogenic responses through Runx2 [30]. Some studies have shown that the pharmacologic induction of mitochondrial stress modulates calcification in the aortic valve. Huibing Liu et al. found that Celastrol alleviates aortic valve calcification by reducing ROS generation in a rabbit model of CAVD and in AVICs [47]. Moreover, in our previous study, 4-Octyl itaconate alleviated OM-induced calcification in vitro and alleviated aortic stenosis in mice with aortic wire injury by reducing ROS production [26]. Furthermore, N-acetyl-L-cysteine (NAC) decreased OM-induced ROS induction and then downregulated ALP and Runx2 expression in human AVICs, suggesting that OM-induced osteogenic differentiation occurs in a ROS-dependent manner. Therefore, targeting mitochondrial stress may be an effective strategy for treating CAVD.

Previous studies have shown that PTPN1 knockout reduces the levels of ROS and augments mitochondrial mass in palmitate-treated oval cells, suggesting that PTPN1 deficiency prevents oxidative stress [3]. However, the role of PTPN22 in mitochondrial dysfunction is unclear. In this study, we found that treatment with PTPN22 siRNA attenuated the overexpression of ROS and enhanced the MMP in human AVICs, providing independent evidence that PTPN22 inhibition protects against mitochondrial dysfunction in human AVICs.

We also discovered that PTPN22 silencing decreased the phosphorylation of NF-κB and ERK signalling, which are well-known pathologic pathways involved in the progression of CAVD [48]. Loss of PTPN22 reduced NF-κB p65 phosphorylation in response to IFN-γ and LPS stimulation [39], which is consistent with our findings. Furthermore, it has been reported that SOCS1 interacts with nuclear NF-κB p65 through polyubiquitination and proteasomal degradation [49]. However, PTPN22 knockdown induces SOCS1 activation and suppresses NF-κB p65 phosphorylation [39]. Here, we found that PTPN22 ablation prevented against mitochondrial stress, which may explain the reduction in NF-κB p65 activation in cells treated with PTPN22 siRNA. Accordingly, the mechanism by which PTPN22 regulates NF-κB and ERK signalling may be involved in mitochondrial stress in AVICs.

Another major finding of this study is the discovery of a novel PTPN22 inhibitor, piericidin diglycoside S18, which was obtained from marine-derived *Streptomyces* [34]*.* The present study demonstrated that piericidin diglycoside S18 bound to the active site residues of PTPN22 through hydrogen bonds. Consistent with the binding relationship with PTPN22, we found that piericidin diglycoside S18 decreased the protein and mRNA levels of PTPN22, suggesting its potent inhibitory effect on PTPN22. Furthermore, piericidin diglycoside S18 not only alleviated OM-induced osteogenic responses of human AVICs but also ameliorated aortic valve thickening and calcium deposition in mice with CAVD. It is worth highlighting that OM-induced mitochondrial stress in human AVICs was significantly attenuated by S18 administration, and as stated previously, S18 could moderate osteogenic responses in AVICs and aortic valve calcification by mitigating mitochondrial dysfunction and excessive ROS production. Thus, S18 is a novel and rare marine-derived compound with therapeutic potential, and in which S18 ameliorates osteogenic differentiation in AVICs and aortic valve lesions in mice in this study. As anticipated, these findings shed light on the therapeutic potential of S18 for CAVD.

There are some limitations in this study. First, we did not establish cell-type-specific gene-modified mice in vivo. Data from PTPN22-knockout mice harbouring a cell-type specific deletion of PTPN22 in human AVICs will be more convincing. Second, given that CAVD is a multifactorial disease and S18 may have multiple targets in CAVD, it cannot be excluded that S18 protects the aortic valve from lesions through other pathways. Collectively, our results indicate that piericidin diglycoside S18 might be a therapeutic candidate for CAVD.

## Conclusions

In conclusion, our study has provided convincing evidence of the essential role of PTPN22 in CAVD. We also show that it is a pathologic feature in CAVD for the upregulation of PTPN22 expression and that PTPN22 aggravates mitochondrial stress to induce osteogenic responses in vitro. We also discovered a novel PTPN22 inhibitor, S18, which attenuates the osteogenic responses of AVICs. Pharmacologic inhibition and genetic ablation of PTPN22 alleviates aortic valve lesions in a well-established mouse model. These novel findings provide a foundation for unveiling the pathogenesis of CAVD and for discovering novel potential medical therapies for the progression of CAVD.

## Supplementary Information

**Additional file 1:** **Additional Figure S1.** The NF-κB and ERK1/2 signalling pathways are involved in the progression of CAVD. **Additional Figure S2.** Piericidin diglycoside S18 inhibits calcification in human AVICs by interfering with NF-κB activation. **Additional Figure S3.** Piericidin diglycoside S18 modulates mitochondrial dysfunction in human AVICs. **Additional Figure S4.** Pathological analysis in a wire injury CAVD mouse model. **Additional Figure S5.** PTPN22 overexpression was detected by western blotting. **Additional Figure S6.** The results of qRT-PCR analysis in gene loss function-associated experiments. **Additional Figure S7.** Alizarin staining of aortic valves after wire injury. **Additional Figure S8.** Alizarin staining of aortic valves after wire injury. **Additional Figure S9.** Diagram shows the animal experimental design. **Additional Table S1.** Clinical characteristics of patients for cultured cells. **Additional Table S2.** Clinical characteristics of patients for transcriptome sequencing. **Additional Table S3.** Primers for qRT‒PCR.

**Additional file 2.**

## Data Availability

The data involved in this article will be shared upon reasonable request to the corresponding author.

## Abbreviations

ALP: Alkaline phosphatase
AVA: Aortic valve area
AVI: Aortic valve injury
AVICs: Aortic valve interstitial cells
BMP2: Bone morphogenetic protein-2
CAVD: Calcific aortic valve disease
DCFH: 2,7-Dichlorodihydrofluorescein diacetate
DUSP: Dual specificity protein phosphatase
GSEA: Gene set enrichment analysis
HFD: High-fat diet
HPLC: High-performance liquid chromatography
HRMS: High-resolution mass spectrometry
LC–MS/MS: Liquid chromatography-tandem mass spectrometry
MMP: Mitochondrial membrane potential
MST: Microscale thermophoresis
NMR: Nuclear magnetic resonance
OM: Osteogenic medium
pCMV3-control: Empty vector
pCMV3-PTPN22: PTPN22 expression vector
PTPN22: Protein tyrosine phosphatase nonreceptor type 22
PTPs: Protein tyrosine phosphatases
qRT-PCR: Quantitative real-time polymerase chain reaction
ROS: Reactive oxygen species
Runx2: Runt-related transcription factor-2
siRNA: Small interfering RNA
TMRM: Tetramethylrhodamine, ethyl ester
