# Supplementary material for: Targeted inhibition of PTPN22 is a novel approach to alleviate osteogenic responses in aortic valve interstitial cells and aortic valve lesions in mice
Source: BMC Med. 2023 Jul 13;21:252. doi: 10.1186/s12916-023-02888-6 (PMC10347738; doi:10.1186/s12916-023-02888-6)
Supplement: Supplementary file 1 — Additional file 1: Additional Figure S1. The NF-κB and ERK1/2 signalling pathways are involved in the progression of CAVD. Additional Figure S2. Piericidin diglycoside S18 inhibits calcification in human AVICs by interfering with NF-κB activation. Additional Figure S3. Piericidin diglycoside S18 modulates mitochondrial dysfunction in human AVICs. Additional Figure S4. Pathological analysis in a wire injury CAVD mouse model. Additional Figure S5. PTPN22 overexpression was detected by western blotting. Additional Figure S6. The results of qRT-PCR analysis in gene loss function-associated experiments. Additional Figure S7. Alizarin staining of aortic valves after wire injury. Additional Figure S8. Alizarin staining of aortic valves after wire injury. Additional Figure S9. Diagram shows the animal experimental design. Additional Table S1. Clinical characteristics of patients for cultured cells. Additional Table S2. Clinical characteristics of patients for transcriptome sequencing. Additional Table S3. Primers for qRT‒PCR. [file 12916_2023_2888_MOESM1_ESM.pdf]

Targeted inhibition of PTPN22 is a novel approach to alleviate osteogenic responses in aortic valve interstitial cells and aortic valve lesions in mice

Shunyi Li [1,2], Zichao Luo [1,2], Shuwen Su [1,2], Liming Wen [1,2], Gaopeng Xian [1,2], Jing Zhao [3], Xingbo Xu [4], Dingli Xu [1,2,*], Qingchun Zeng [1,2,*]

[1] State Key Laboratory of Organ Failure Research, Department of Cardiology, Nanfang Hospital, Southern Medical University, Guangzhou, 510515, China

[2] Guangdong Provincial Key Laboratory of Cardiac Function and Microcirculation, Southern Medical University, Guangzhou,510515, China

[3] State Key Laboratory of Quality Research in Chinese Medicine, Institute of Chinese Medical Sciences, University of Macau, Macau, China

[4] Department of Cardiology and Pneumology, University Medical Center of Göttingen, Georg-August-University, Göttingen, Germany

*Corresponding authors. E-mail: dinglixu@smu.edu.cn (D.X.); qingchunzeng@smu.edu.cn (Q.Z.)

1. Methods and Materials

1.1 Cell Viability Analysis and CCK8 Assay

Cell viability was analyzed using CKK-8 assay kits (Glpbio, GK10005, USA) according to the manufacturer's instructions. Human AVICs were cultured in each well of a 96-well plate in M199 growth medium supplemented with 10% FBS for 24 hours. Then, the cells were starved in serum-free medium for 12 hours. Subsequently, the cells were treated with different final concentrations of S18 (0 - 4 μM) for 72 hours. CCK8 solution was added to each well of the plate according to the manufacturer's instructions, and the plate was incubated in an incubator with 5% CO_2 at 37°C for 2 hours. The absorbance of the samples was determined spectrophotometrically at 450 nm using a microplate reader.

1.2 Transcriptome Sequencing

Aortic valve from CAVD patients and non-CAVD patients were performed for transcriptome sequencing as described previously [33]. Briefly, the RNA of aortic valve was isolated through Trizol reagent. And the quality control of RNA sample was based on qualified ratio of OD260-280. Then, RNA sample preparation was used by CloudSeq Biotech (Shanghai, CA). The RNA libraries were constructed with TruSeq Stranded Total RNA Library Prep Kit (Illumina, San Diego, CA, USA) according to the manufacturer's instructions. The DeSeq2 software packages for the R programming environment and quantile algorithm were performed for quantile normalization and subsequent data processing. Significantly differentially expressed transcripts were screened by fold change ≥ 1.5 or ≤ -1.5 and P value ≤ 0.05.

1.3 Immunoblotting

Immunoblotting was applied to analyze the levels of ALP (ABconal, A0514, 1:2000 dilution, CN), Runx2 (Abcam, Ab23981, 1:1000 dilution, CN), BMP2 (ProSci, XP-5111 , 1:800 dilution, USA), PTPN22 (ABconal, A1406, 1:800 dilution, CN), phosphorylated NF-κB p65(Cell Signaling Technology, #3033, 1:800 dilution, USA), total NF-κB p65 (Cell Signaling Technology, #8242, 1:1000 dilution, USA) levels, phosphorylated ERK1/2 (Cell Signaling Technology, #9101, 1:2000 dilution, USA), total ERK1/2 (Cell Signaling Technology, #4695, 1:2000 dilution, USA), GAPDH (Protechtein, 60004-1-Ig, 1: 8000 dilution, USA) and β-actin (Protechtein, 66009-1-Ig, 1:8000 dilution, USA) in human

AVICs.

Briefly, cells and tissue were lysed in a lysis buffer (Solarbio, R0010, CN) containing phosphatase and protease inhibitors (Solarbio, P6730, P1260, CN) and quantified with a bicinchoninic acid protein assay. The protein samples were separated using SDS-PAGE gels and transferred into PVDF membranes (EMD Millipore, Billerica, MA, USA). After blocking with 5% skim milk in TBST solution (Boster, AR0031, CN) at room temperature for 1 hour, the blocked membranes were incubated overnight at 4°C with primary antibodies. After washing with TBST, the membranes were incubated with an HRP-conjugated secondary antibody specific to the primary antibodies for 1 hour at room temperature. GAPDH and β-actin were analyzed for normalizing protein loading. In the phosphorylation assay, total NF-κB and ERK1/2 were used for normalization. Finally, immunoreactive bands were detected with FDbio-Dura ECL Kit (Fdbio Science, FD8020, CN) and GeneGnome imaging system (Syngene, Frederick, MD, USA). The images were analyzed with ImageJ software.

1.4 Alizarin Red S Staining and Von Kossa Staining

Alizarin red S staining was performed as described as previously to detect the levels of calcium deposits [23]. Briefly, after treatment, cells were washed with PBS, then fixed in 4% paraformaldehyde for 15 minutes at room temperature. After washing with PBS, cells were incubated with 0.2 % alizarin red solution (pH 4.2) for 15 minutes at room temperature. The images were acquired with OLYMPUS CKX41 microscope.

Calcium deposition staining was stained using Von Kossa Kit (Servicebio, G1042, CN) in mouse aortic valve tissue according to the manufacturer's protocol. Calcium depositions were evaluated in vivo using ImageJ software.

1.5 RNA Purification and Quantitative Real-time Polymerase Chain Reaction

Total RNA was extracted from human AVICs using EZ-press RNA Purification Kit (EZBioscience, B004D, USA) following the manufacturer's instructions. The cDNA for each sample was reverse transcribed using the EVO M-MLV First Strand cDNA Synthesis Kit (Accurate Biotechnology (Hunan)Co.,Ltd, AG11706, CN). PCR was performed using SYBR Green premix Pro Taq HS qPCR Kit (Accurate Biotechnology (Hunan)Co.,Ltd, AG11701, CN) and LightCycler 480 (Roche Diagnostics, Basel, Switzerland). The following primers were used to amplify specific cDNA fragments. The mean relative gene

expression was calculated with the $2^{-\Delta\Delta Ct}$ method after normalization of the cycle threshold (Ct) values to GAPDH gene expression (Table S3).

1.6 Hematoxylin and eosin (H&E) staining and Masson staining

H&E staining was employed to detect the thickness of mice aortic valves using H&E Kit (Solarbio, G1120, CN), according to the manufacturer's protocol. The thickness of aortic valves is the average value of the three thickest leaflets in six sections each mouse[23].

Masson staining was employed to detect the level of collagen in mice aortic valves using Masson staining Kits (Solarbio, G1340, CN), according to the manufacturer's protocol. The fractional area of collagen fibrosis components (blue) in the aortic valve was acquired ImageJ 1.55 (National Institutes of Health)[14].

1.7 Immunofluorescence staining

Immunofluorescence staining was employed to detect ALP, Runx2, BMP2, and NF-κB, levels in human AVICs or aortic valves of mice. After cell treatment, human AVICs were rinsed briefly with PBS, fixed in 4% paraformaldehyde for 15 minutes, and permeabilized with PBS containing 0.3% Triton X-100 for 10 minutes at room temperature. Nonspecific immunoreactions were blocked using 5% BSA in PBS for 1 hour at room temperature. Human AVICs were incubated overnight with primary antibodies. After secondary antibody (Proteintech, SA00013-2, 1:300 dilution, CN) incubation, the cell nuclei were stained with DAPI (Solarbio, C0060, CN) for 15 minutes, and then microscopy images were visualized using Leica TCS SP8.

A similar protocol was performed to stain the tissue sections. The mouse valves and human valves were deparaffinized with xylene and grades of ethanol, followed by antigen retrieval. After permeabilization and blocking with 5% BSA for 1 hour, the sections were incubated with primary antibodies. After washing with PBS, the sections were incubated with secondary antibodies conjugated to Alexa Fluor 552 (Proteintech, SA00009-1, SA00009-2, 1:300 dilution, CN). Cell nuclei were stained with DAPI for 15 minutes, and images were acquired using a Leica TCS SP8.

1.8 ROS and mitochondrial membrane potential (MMP) detection

ROS levels were detected using DCFH-DA Kit (Beyotime, S0033S, CN) as described in the manufacturer's protocol. The MMP was determined using the TMRM Kit (Beyotime,

C2001S, CN) following the manufacturer's instructions. After treatment, cells were cultured with TMRM staining solution for 30 minutes at 37 °C. The images were acquired using a Leica TCS SP8 and were analyzed using ImageJ software.

1.9 Fermentation and isolation of S18.

The strain Streptomyces psammoticus SCSIO NS126, derived from a mangrove sediment sample collected from the Pearl River estuary of the South China Sea, was fermented in a volume of 100 L, according to a previously described method[34]. The culture broth was extracted with ethyl acetate three times and then concentrated under a vacuum. The extract (75.0 g) underwent chromatography on silica gel to give eight fractions (Frs. 1~8). With the guidance of high-performance liquid chromatography (HPLC) analysis, Frs. 7 was purified with silica gel again to give six subfractions (Frs. 7-1~7-6). S18 (58 mg) was obtained and purified by repeated semipreparative HPLC isolation. The obtained compound was determined to be ≥ 95% purity by analytical HPLC. The structures of the obtained compounds were determined to be S18 by comparison of high-resolution mass spectrometry (HRMS) and nuclear magnetic resonance (NMR) data with literature data. The compounds were stored at -20°C until use and dissolved in DMSO to a stock concentration of 10 mM.

1.10 Molecular docking.

The Schrödinger 2017-1 suite (Schrödinger Inc., New York, USA) was employed to perform the docking analysis, according to a previously described method[34]. The PTPN22 structure was retrieved from the available crystal structures (PDB: 4J51) and constructed following the protein Preparation Wizard workflow in the Maestro package. The binding site was selected using the Grid Generation procedure. The prepared ligand was flexibly docked into the receptor using Glide (XP mode) with default parameters.

1.11 Microscale thermophoresis (MST)

The ability of piericidin diglycosides S18 to bind with potential ligands was analysed using MST. Monolith NT.115 instrument (Nano Temper Technologies, Germany) was employed. The cell lysates of HEK293T cells expressing GFP-PTPN22 were the source of fluorescently labeled PTPN22. Cells were transfected with PTPN22 expression vector (pCMV3-C-GFP-PTPN22, SinoBiological, HG12626-ACG, CN). The cell lysates were diluted 10× times with MST buffer to provide an optimal level of fluorescence. The lysates

of cells transfected with control vector (pCMV3-C-GFP, SinoBiological, CV026, CN) were performed as a control. The compounds were diluted in 16 dilution steps covering the range of appropriate concentrations. After 15 minutes of incubation at room temperature, the samples were loaded into capillaries, and thermophoresis was measured on Monolith NT.115 instrument with 50% infrared-laser power. The K_d model was used to detect binding affinity and the K_d value was calculated using NT Analysis software (Nano Temper Technologies, Germany).

2. Supplementary Figure Legends

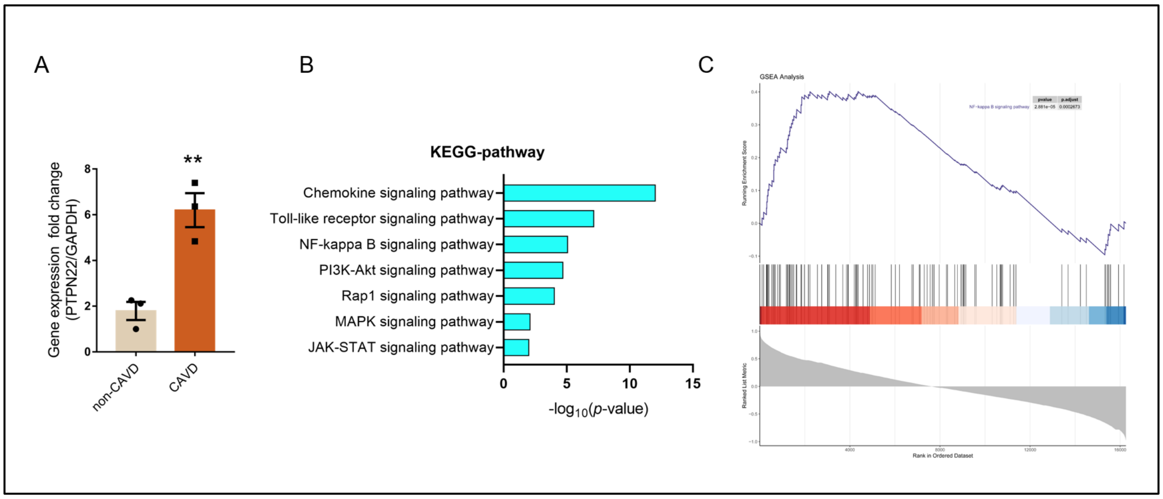

Additional Figure S1. NF-κB and ERK1/2 signaling pathways are involved in the progression of CAVD. (A)The PTPN22 overexpression was measured by qPCR analysis in aortic valve. n = 3. *p<0.05 (B) KEGG (Kyoto Encyclopedia of Genes and Genomes) pathway analysis for genes sets is involved in CAVD. (C) Gene set enrichment analysis (GSEA) for the gene sets involved in NF-κB signaling pathway on the transcriptome data from aortic valve.

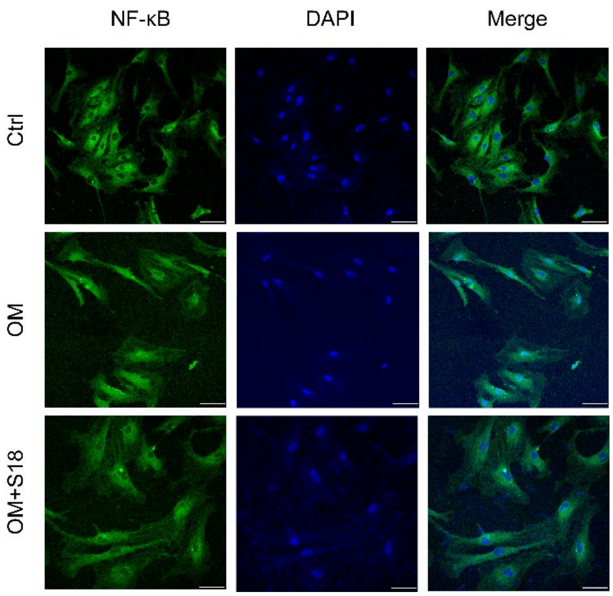

Additional Figure S2. Piericidin diglycoside S18 inhibits calcification in human AVICs via interfering with NF-κB activation. Cells were cultured in OM and treat with or without S18. (A) Immunofluorescence staining show intranuclear localization of NF-κB p65 in human AVICs simulated with OM, and extranuclear localization after human AVICs treated with S18. Scale bar = 100 μm.

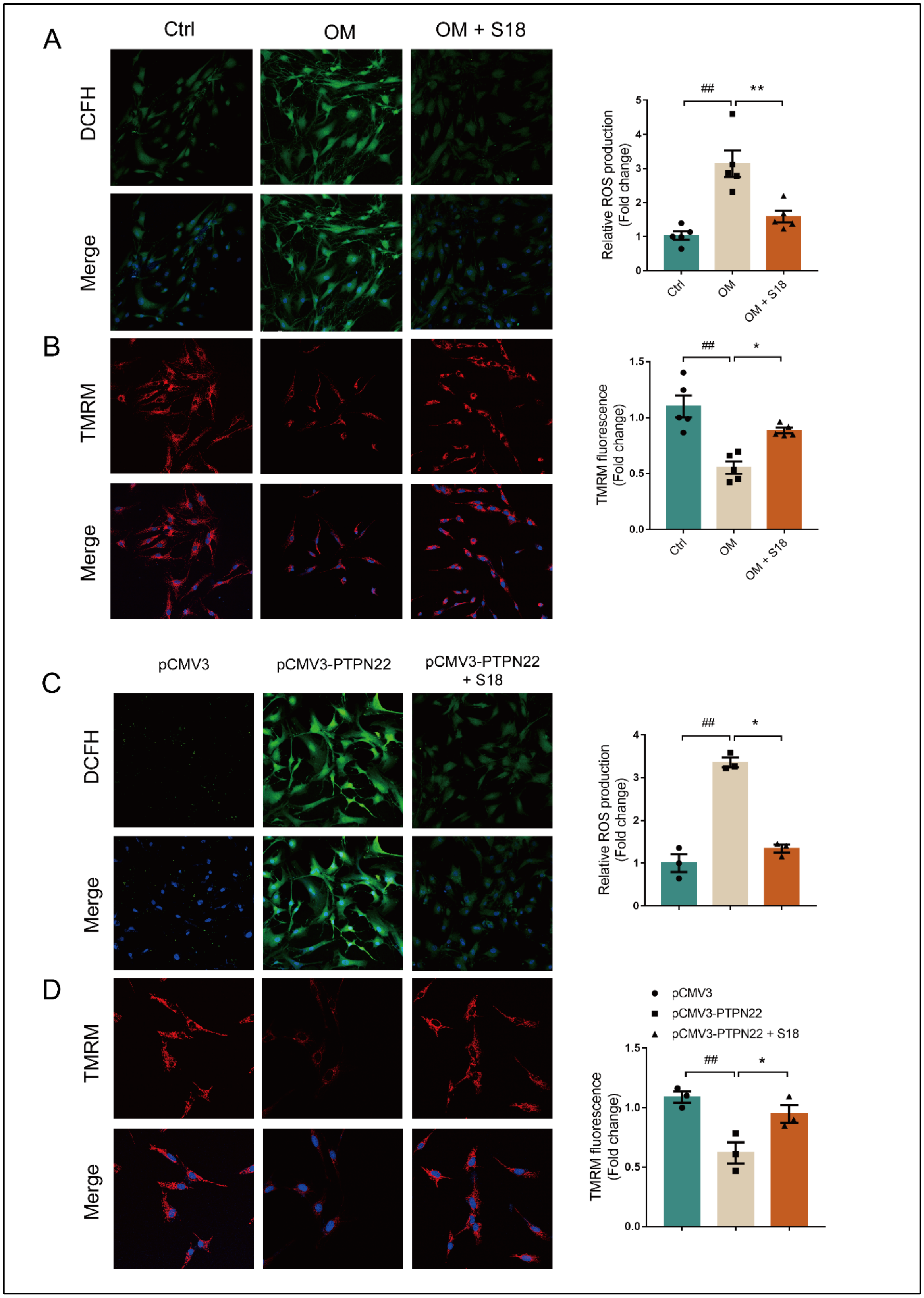

Additional Figure S3. Piericidin diglycoside S18 modulates mitochondrial dysfunction in human

AVICs. (A) Fluorescence images using DCFH-DA fluorescent dye show ROS production, n = 5. Scale bar = 50 μm. (B) Fluorescence intensity of TMRM staining, n = 5. Scale bar = 50 μm. Value are the means ± SEM ##$p < 0.01$ vs. the ctrl group, *$p < 0.05$, **$p < 0.01$ vs. the OM group. Cells were transfected with control or pCMV3-PTPN22 prior to treatment with S18. (C) DCFH-DA fluorescence images showing ROS production, n = 3. Scale bar = 50 μm. (D) Fluorescence intensity of TMRM staining, n = 3. Scale bar = 25 μm. Value are the means ± SEM. #$p < 0.05$, ##$p < 0.01$ vs. the pCMV3 group, *$p < 0.05$, **$p < 0.01$ vs. the pCMV3-PTPN22 group.

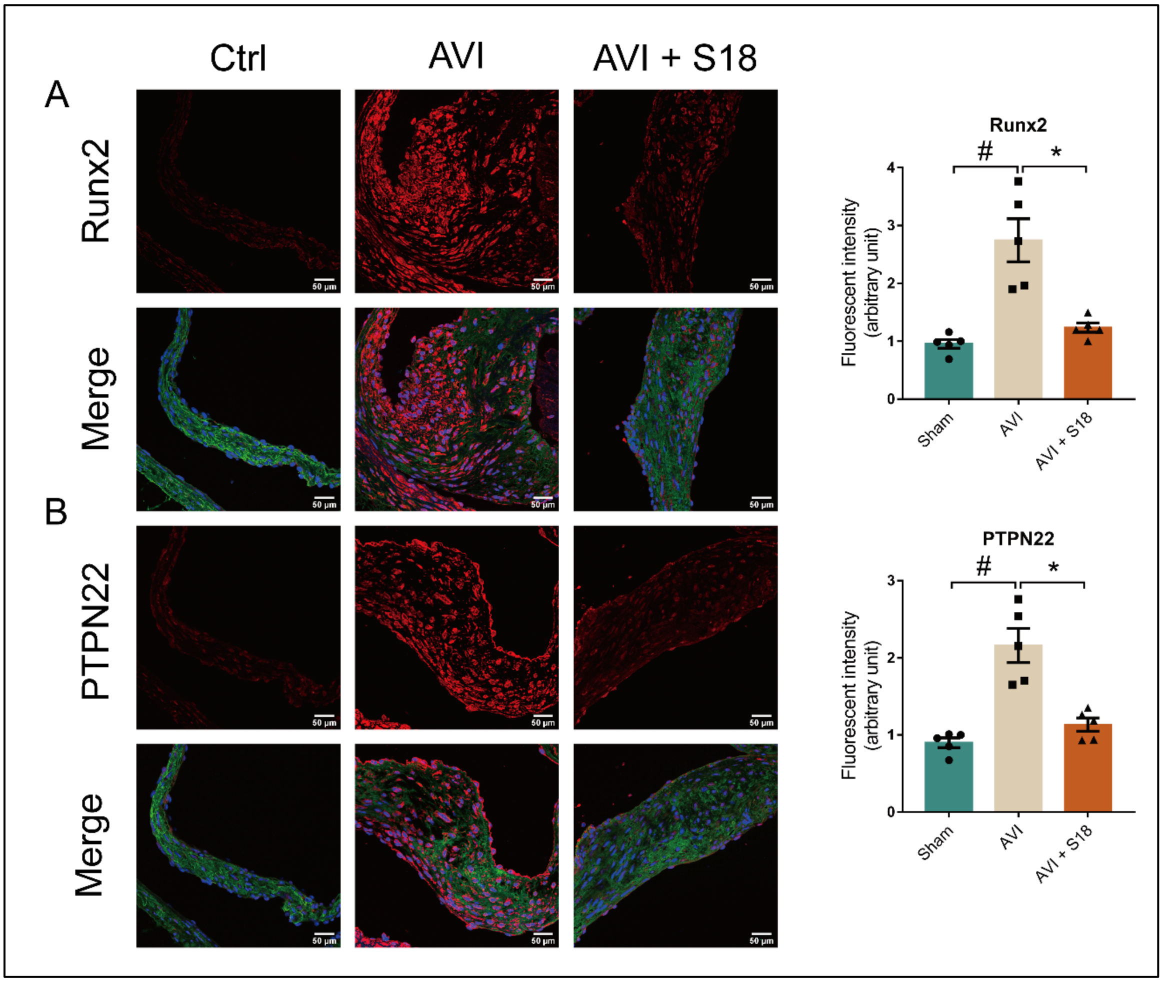

Additional Figure S4. Pathological analysis in in wire injury CAVD model mice. Representative micrographs show the immunofluorescence staining of Runx2, and PTPN22 expression in murine aortic valve at 12 weeks after wire injury, n = 5. Scale bar = 50 µm. Error bars represent means ± SEM. #*p*< 0.05, ##*p*< 0.01 vs. the sham group, **p*< 0.05, ***p*< 0.01 vs. the AVI + S18 group.

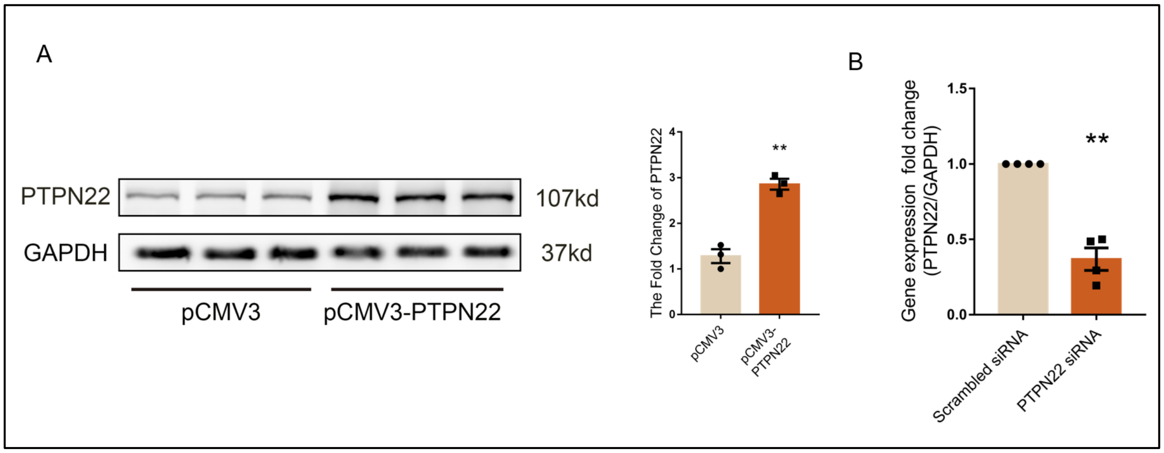

Additional Figure S5. (A)The PTPN22 overexpression was measured by western blotting analysis in AVICs transfected with pCMV3-PTPN22. n = 3. *p<0.05. (B) The PTPN22 siRNA was detected by qPCR analysis in AVICs. n = 4. **p<0.01

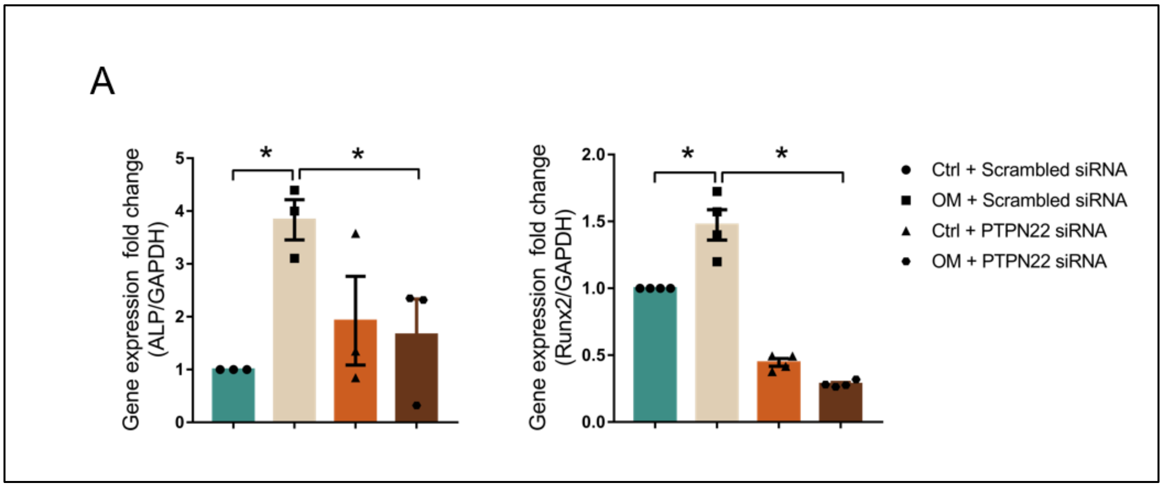

Additional Figure S6. (A) Cells were treated with PTPN22 siRNA following stimulation with OM. Representative qPCR on the relative abundance of ALP, and Runx2 in the different groups. n = 3 or 4. Values are the means ± SEM. *p< 0. 5.

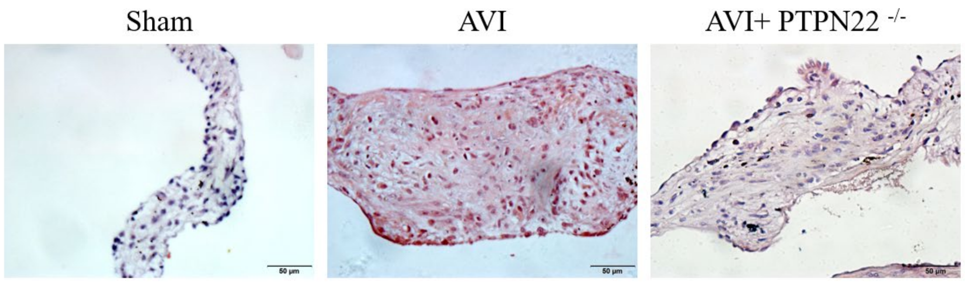

Additional Figure S7. Pathological analysis in in wire injury CAVD model mice. Image of aortic valves in the aortic valve calcification model. Alizarin staining of aortic valves after wire injury. Scale bar = 50 μm.

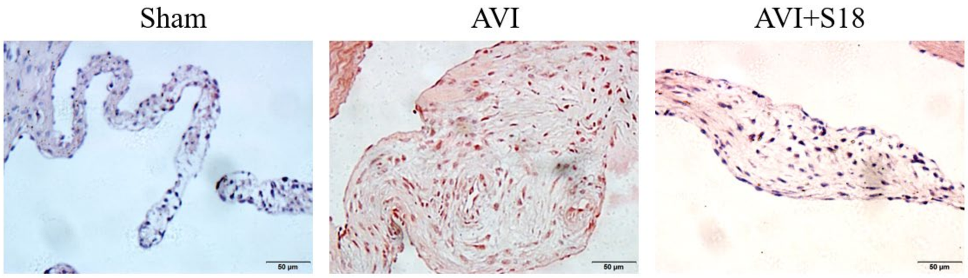

Additional Figure S8. Pathological analysis in in wire injury CAVD model mice. Image of aortic valves in the aortic valve calcification model. Alizarin staining of aortic valves after wire injury. Scale bar = 50 μm.

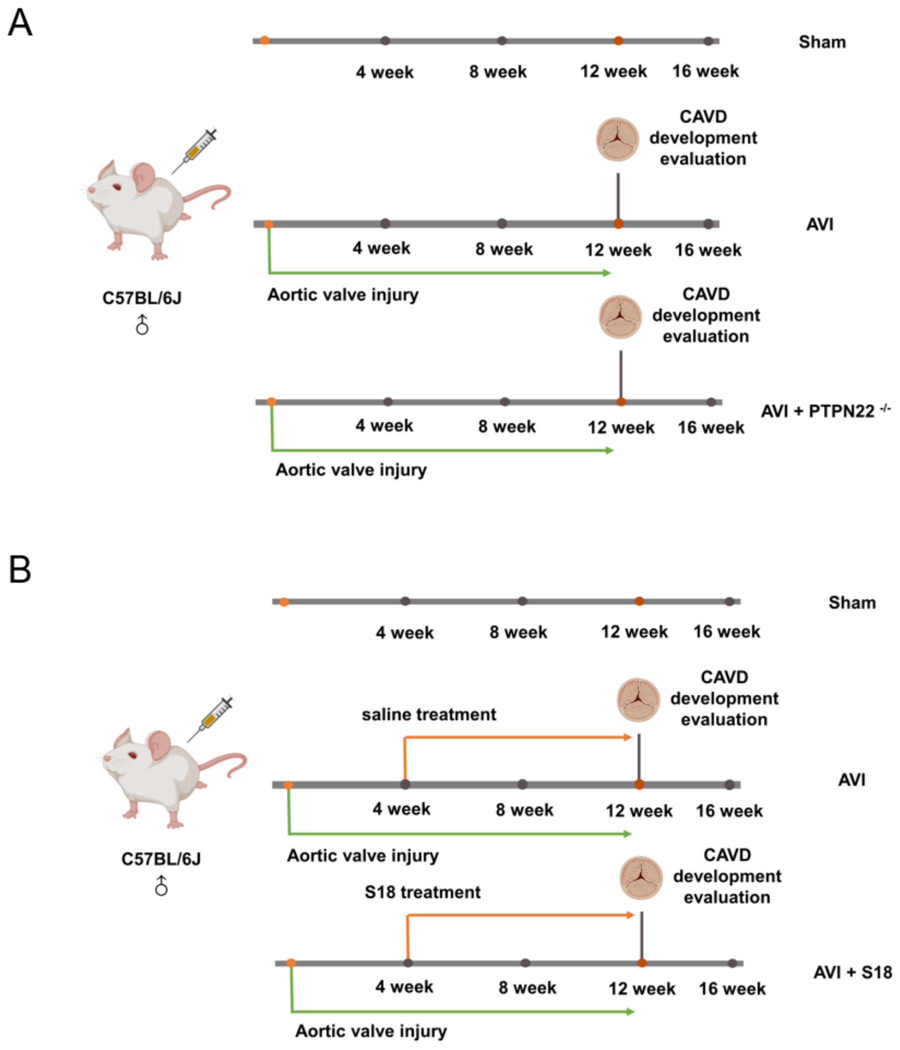

Additional Figure S9. Diagram shows the animal experimental design.

9. Additional Table S1. Clinical characteristics of patients for cultured cells

	Non-CAVD patients (n=6)	CAVD patients (n=6)
Age (years)	58.0 ± 4.79	66.6 ± 6.04
Male, n (%)	4 (67%)	3 (50%)
BMI (Kg/m^2)	28.2 ± 1.34	25.9 ± 1.69
Hypertension, n (%)	3 (50%)	3 (50%)
Dyslipidaemia, n (%)	2 (33%)	4 (67%)
Diabetes mellitus, n (%)	4 (67%)	3 (50%)
Smoking history, n (%)	4 (67%)	2 (33%)
β-blockers, n (%)	3 (50%)	5 (83%)
ACE inhibitors/ARB, n (%)	3 (50%)	4 (67%)
Statins, n (%)	2(33%)	4 (67%)
LVEF (%)	65.4 ± 6.72	60.8 ± 5.73
AO (cm/s)	-	388.4 ± 40.31

Data are presented as mean ± SEM, n (%), or median (interquartile range). ACE, angiotensin converting enzyme; ARB, angiotensin receptor blocker; AO, aorta valve peak flow velocity; BMI, body mass index; CAVD, calcific aortic valve disease; LVEF, left ventricle Ejection fraction;

10. Additional Table S2. Clinical characteristics of patients for transcriptome sequencing

	Non-CAVD patients (n=3)	CAVD patients (n=3)
Age (years)	56.6 ± 5.89	65.87 ± 6.04
Male, n (%)	2 (67%)	1 (33%)
BMI (Kg/m^2)	29.34 ± 1.64	26.56 ± 1.89
Hypertension, n (%)	2 (67%)	3 (100%)
Dyslipidaemia, n (%)	2 (67%)	2 (67%)
Diabetes mellitus, n (%)	1 (33%)	3 (100%)
Smoking history, n (%)	1 (33%)	2 (67%)
β-blockers, n (%)	2 (67%)	3 (100%)
ACE inhibitors/ARB, n (%)	2 (67%)	3 (100%)
Statins, n (%)	2 (67%)	2 (67%)
LVEF (%)	53.4 ± 4.72	59.8 ± 4.93
AO (cm/s)	-	368.4 ± 33.71

Data are presented as mean ± SEM, n (%), or median (interquartile range). ACE, angiotensin converting enzyme; ARB, angiotensin receptor blocker; AO, aorta valve peak flow velocity; BMI, body mass index; CAVD, calcific aortic valve disease; LVEF, left ventricle Ejection fraction;

11. Additional Table S3. Primes for qRT-PCR

Species	Gene name		Primer (5'- 3')
human	ALP	F	ACCACCACGAGAGTGAACCA
		R	CGTTGTCTGAGTACCAGTCCC
	Runx2	F	TGGTTACTGTCATGGCGGGTA
		R	TCTCAGATCGTTGAACCTTGCTA
	PTPN22	F	AGGCAGACAAAACCTATCCTACA
		R	TGGGTGGCAATATAAGCCTTG
	GAPDH	F	AATGGGCAGCCGTTAGGAAA
		R	AATGGGCAGCCGTTAGGAAA
