# Supplementary material for: Targeted inhibition of PTPN22 is a novel approach to alleviate osteogenic responses in aortic valve interstitial cells and aortic valve lesions in mice
Source: BMC Med. 2023 Jul 13;21:252. doi: 10.1186/s12916-023-02888-6 (PMC10347738; doi:10.1186/s12916-023-02888-6)
Supplement: Supplementary file 2 — Additional file 2. [file 12916_2023_2888_MOESM2_ESM.pdf]

Additional file 2

Targeted inhibition of PTPN22 is a novel approach to alleviate osteogenic responses in aortic valve interstitial cells and aortic valve lesions in mice

Shunyi Li [1, 2], Zichao Luo [1, 2], Shuwen Su [1, 2], Liming Wen [1, 2], Gaopeng Xian [1, 2], Jing Zhao [3], Xingbo Xu [4], Dingli Xu [1, 2, *], Qingchun Zeng [1, 2, *]

[1] State Key Laboratory of Organ Failure Research, Department of Cardiology, Nanfang Hospital, Southern Medical University, Guangzhou, 510515, China

[2] Guangdong Provincial Key Laboratory of Cardiac Function and Microcirculation, Southern Medical University, Guangzhou,510515, China

[3] State Key Laboratory of Quality Research in Chinese Medicine, Institute of Chinese Medical Sciences, University of Macau, Macau, China

[4] Department of Cardiology and Pneumology, University Medical Center of Göttingen, Georg-August-University, Göttingen, Germany

*Corresponding authors. E-mail: dinglixu@smu.edu.cn (D.X.); qingchunzeng@smu.edu.cn (Q.Z.)

Figure 1

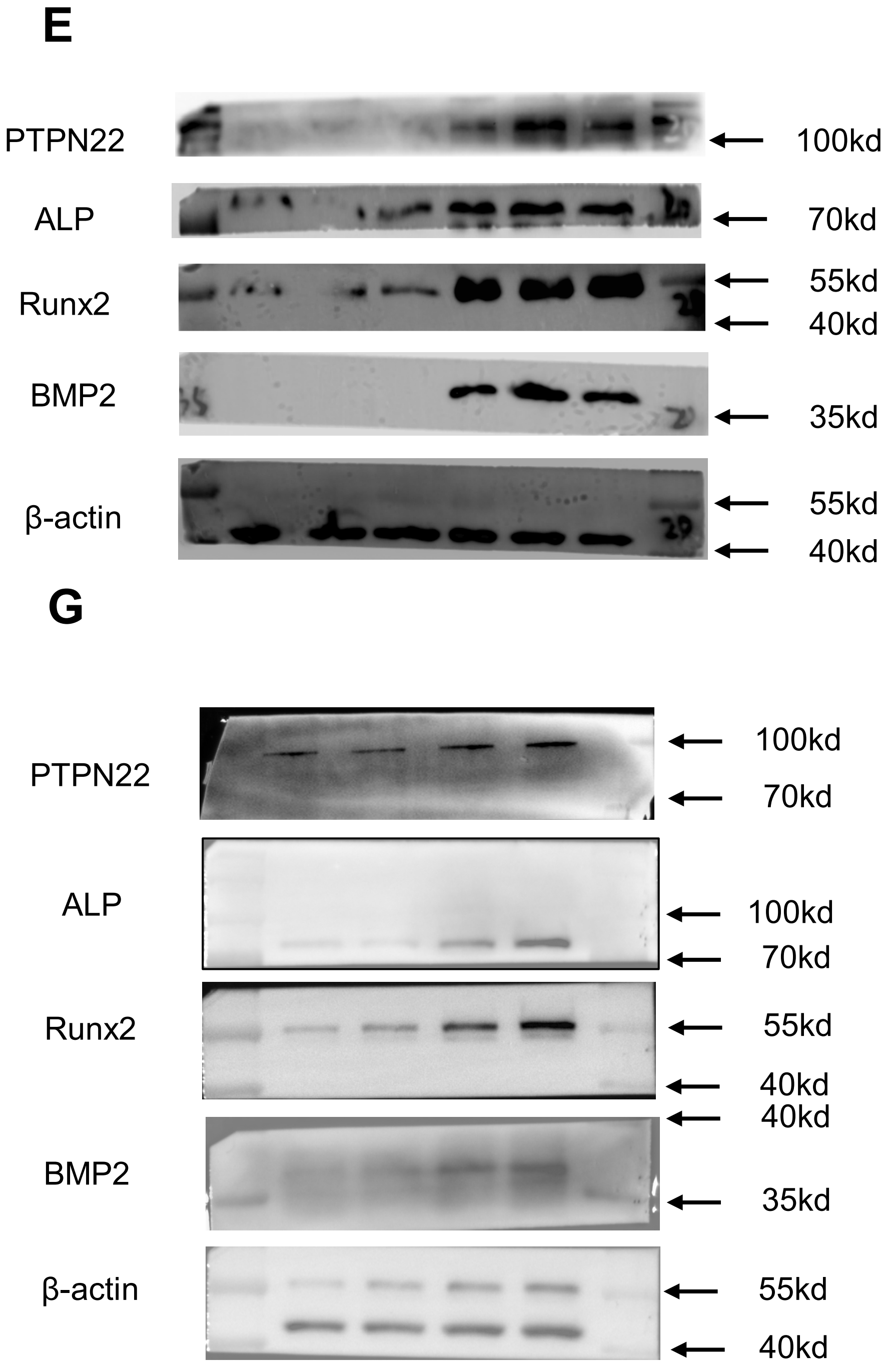
E
PTPN22
100kd
ALP
70kd
Runx2
55kd
40kd
BMP2
35kd
β-actin
55kd
40kd
G
PTPN22
100kd
70kd
ALP
100kd
70kd
Runx2
55kd
40kd
BMP2
40kd
35kd
β-actin
55kd
40kd

Figure 2

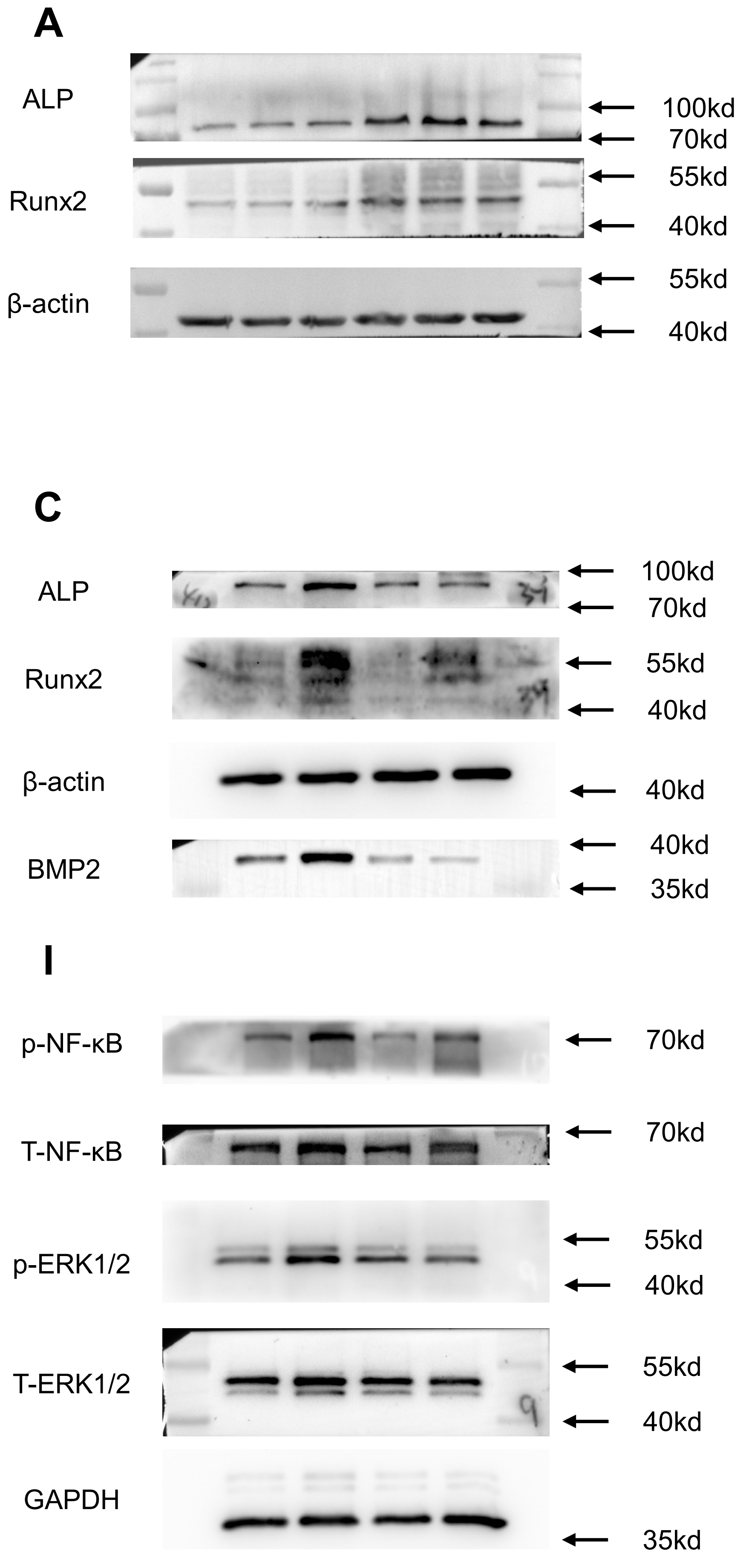
A
ALP
100kd
70kd
Runx2
55kd
40kd
β-actin
55kd
40kd
C
ALP
100kd
70kd
Runx2
55kd
40kd
β-actin
40kd
BMP2
40kd
35kd
I
p-NF-κB
70kd
T-NF-κB
70kd
p-ERK1/2
55kd
40kd
T-ERK1/2
55kd
40kd
GAPDH
35kd

Figure 5

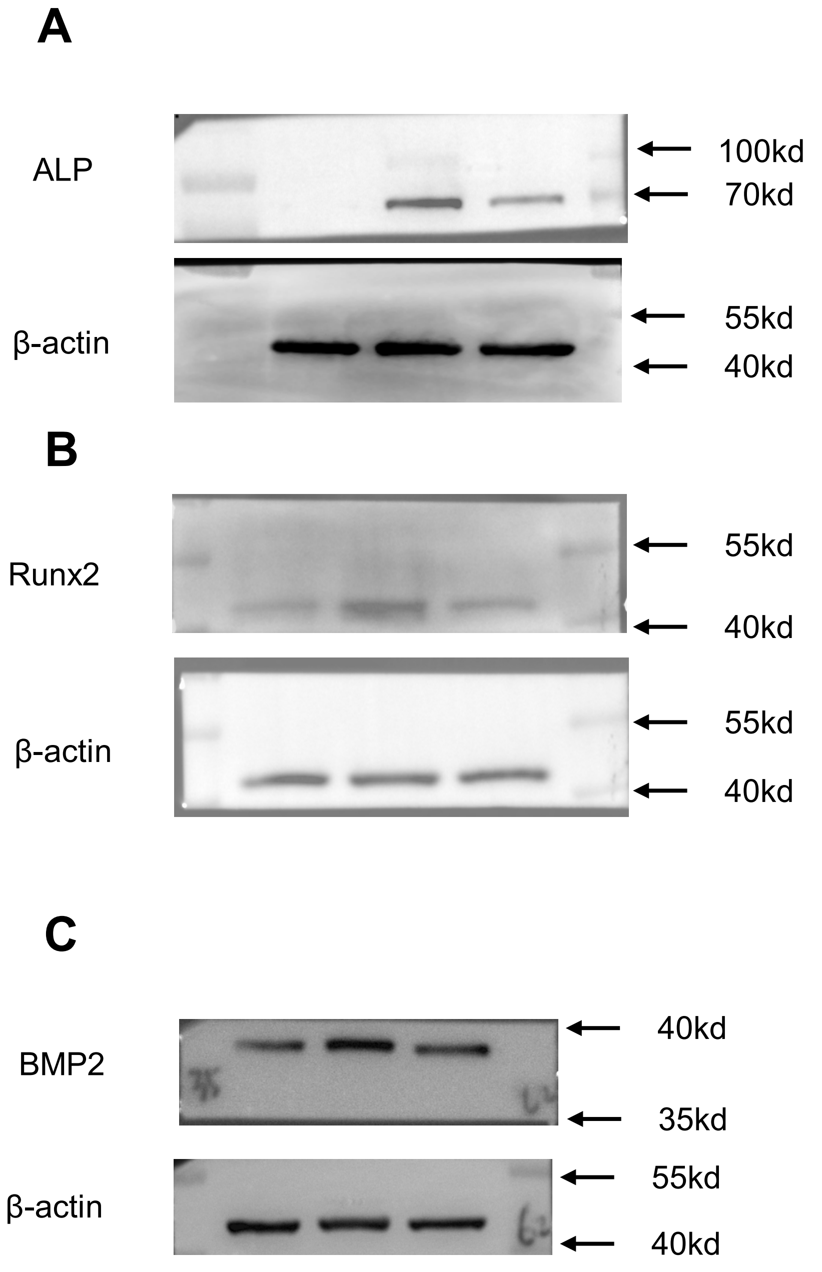

A
ALP
100kd
70kd
β-actin
55kd
40kd
B
Runx2
55kd
40kd
β-actin
55kd
40kd
C
BMP2
40kd
35kd
β-actin
55kd
40kd

Figure 6

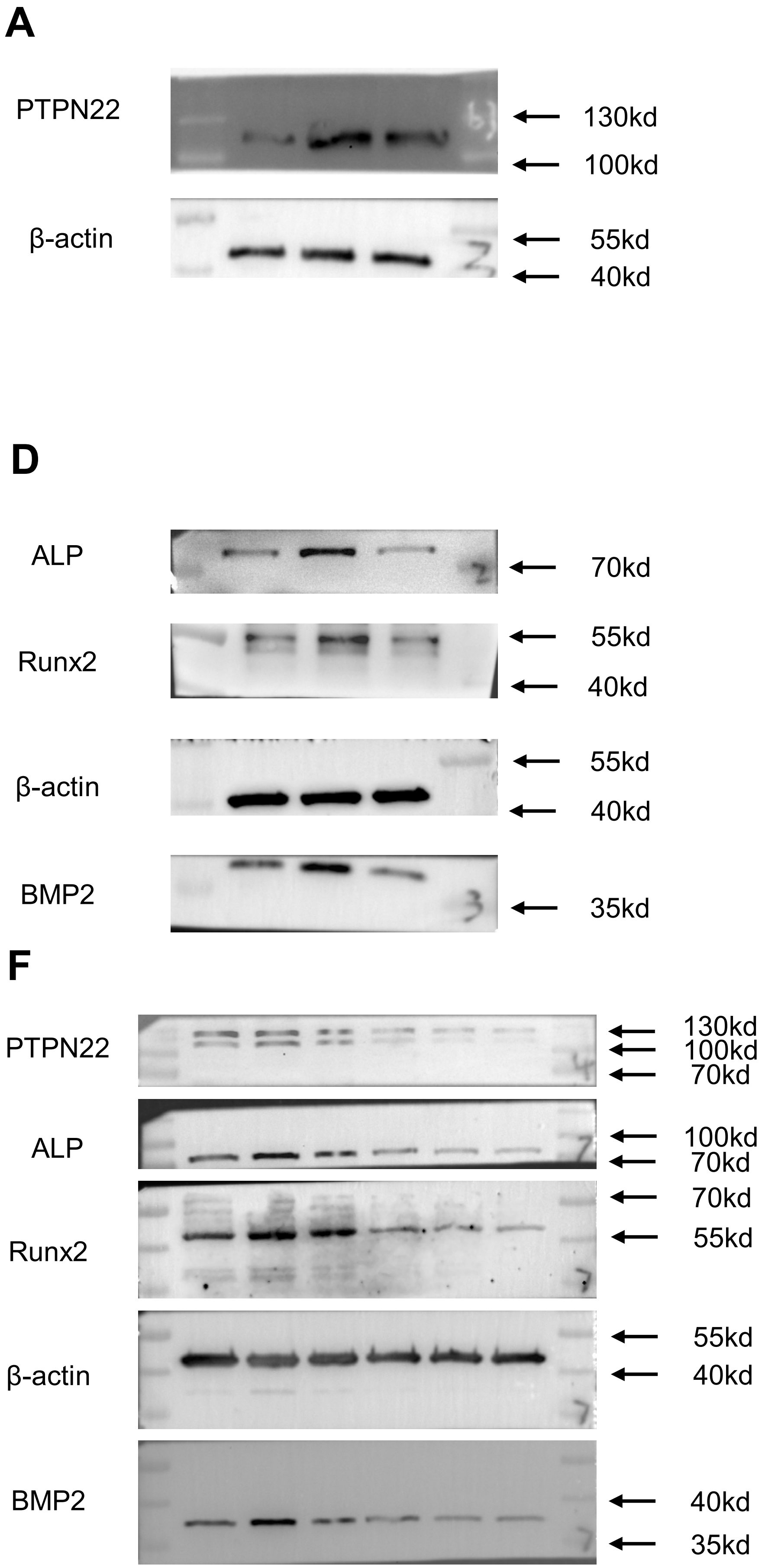
A
PTPN22
130kd
100kd
β-actin
55kd
40kd
D
ALP
70kd
Runx2
55kd
40kd
β-actin
55kd
40kd
BMP2
35kd
F
PTPN22
130kd
100kd
70kd
ALP
100kd
70kd
Runx2
70kd
55kd
β-actin
55kd
40kd
BMP2
40kd
35kd

Figure 6

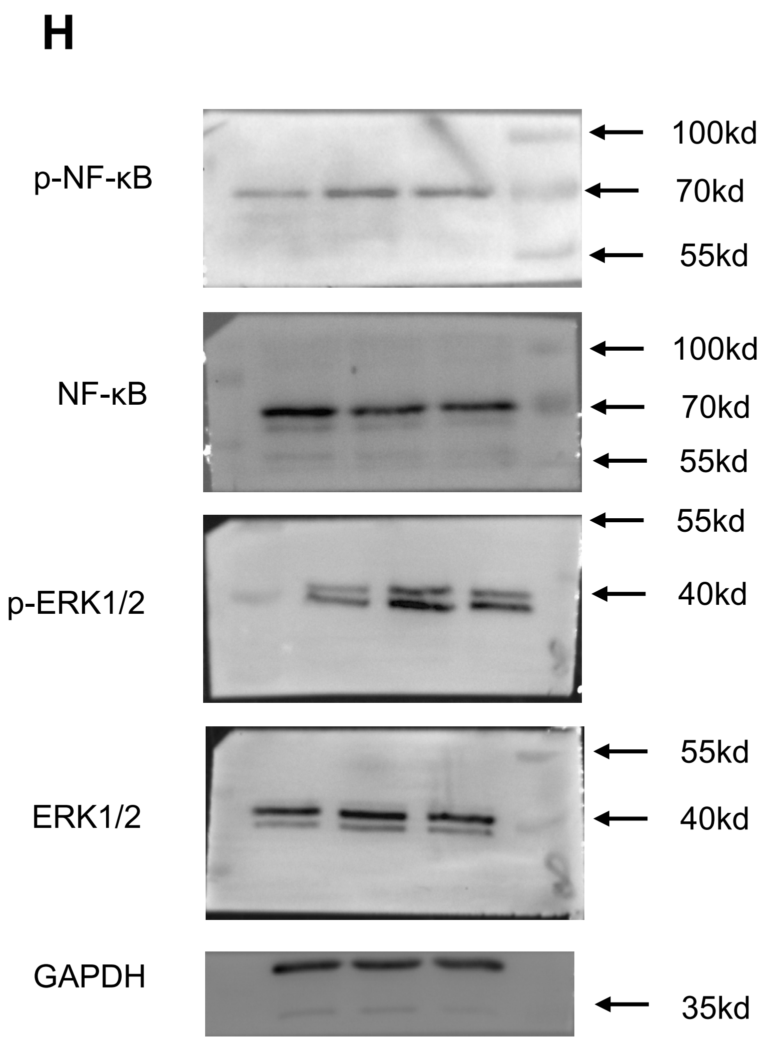
H
p-NF-κB
100kd
70kd
55kd
NF-κB
100kd
70kd
55kd
p-ERK1/2
55kd
40kd
ERK1/2
55kd
40kd
GAPDH
35kd
